# Non-collinear Hox gene expression in bivalves and the evolution of morphological novelties in mollusks

**DOI:** 10.1038/s41598-021-82122-6

**Published:** 2021-02-11

**Authors:** David A. Salamanca-Díaz, Andrew D. Calcino, André L. de Oliveira, Andreas Wanninger

**Affiliations:** 1 Department of Evolutionary Biology, Unit for Integrative Zoology, University of Vienna, Althanstraße 14, 1090 Vienna, Austria; 2 Department of Functional and Evolutionary Ecology, Unit for Bio-Oceanography and Marine Biology, University of Vienna, Althantraße 14, 1090 Vienna, Austria

**Keywords:** Developmental biology, Evolution, Zoology

## Abstract

Hox genes are key developmental regulators that are involved in establishing morphological features during animal ontogeny. They are commonly expressed along the anterior–posterior axis in a staggered, or collinear, fashion. In mollusks, the repertoire of body plans is widely diverse and current data suggest their involvement during development of landmark morphological traits in Conchifera, one of the two major lineages that comprises those taxa that originated from a uni-shelled ancestor (Monoplacophora, Gastropoda, Cephalopoda, Scaphopoda, Bivalvia). For most clades, and bivalves in particular, data on Hox gene expression throughout ontogeny are scarce. We thus investigated Hox expression during development of the quagga mussel, *Dreissena rostriformis*, to elucidate to which degree they might contribute to specific phenotypic traits as in other conchiferans. The Hox/ParaHox complement of Mollusca typically comprises 14 genes, 13 of which are present in bivalve genomes including *Dreissena*. We describe here expression of 9 Hox genes and the ParaHox gene *Xlox* during *Dreissena* development. Hox expression in *Dreissena* is first detected in the gastrula stage with widely overlapping expression domains of most genes. In the trochophore stage, Hox gene expression shifts towards more compact, largely mesodermal domains. Only few of these domains can be assigned to specific developing morphological structures such as *Hox1* in the shell field and *Xlox* in the hindgut. We did not find traces of spatial or temporal staggered expression of Hox genes in *Dreissena*. Our data support the notion that Hox gene expression has been coopted independently, and to varying degrees, into lineage-specific structures in the respective conchiferan clades. The non-collinear mode of Hox expression in *Dreissena* might be a result of the low degree of body plan regionalization along the bivalve anterior–posterior axis as exemplified by the lack of key morphological traits such as a distinct head, cephalic tentacles, radula apparatus, and a simplified central nervous system.

## Introduction

Mollusca constitutes a metazoan phylum with unique morphological diversity. It is composed of two clades, Aculifera and Conchifera, that diverged from one another in the early Cambrian. The Aculifera includes the vermiform, spicule-bearing Solenogastres (Neomeniomorpha) and Caudofoveata (Chaetodermomorpha) as well as the dorso-ventrally flattened Polyplacophora with eight shell plates, while the primarily single-shelled Conchifera contains the Monoplacophora, Scaphopoda, Gastropoda, Bivalvia, and Cephalopoda^1^. Hox genes are key developmental regulators that are involved in specifying morphological regions of the body plan during animal ontogeny^2–6^. One of the central roles of Hox gene products is to define body regionalization along the anterior–posterior axis^2,7,8^. The Hox genes are usually arranged in close proximity to each other on the genome, and this micro-syntenic block is also referred to as the Hox cluster. There is often a correlation between a given Hox gene's relative chromosomal position, its relative spatial expression domain along the animal’s anterior–posterior axis (spatial collinearity), and/or its temporal activation (temporal collinearity)^9–11^. A three-fold collinearity encompassing positional arrangement on the genome as well as tempo-spatial expression is commonly believed to be ancestral to bilaterian animals^12^. However, the recent increase in high quality genome assemblies as well as expression analyses on previously neglected clades have shown that numerous deviations from this model are found in almost all major bilaterian lineages^13–19^.

In Mollusca, 11 Hox genes have been identified^14,16,18,20–38^. While no Hox expression data are available for the two aplacophoran clades, Neomeniomorpha and Chaetodermomorpha, staggered spatial expression of 10 Hox genes along the anterior–posterior body axis has been found in the polyplacophoran *Acanthochitona*^30,31^. This is different from the situation in the conchiferans, where Hox genes are expressed during development of distinct morphological features such as ganglia, the foot, or the shell field^39^. In the gastropod *Gibbula*, *Lox5, Hox7, Lox4*, and *Lox2* are expressed in the larval swimming device, the prototroch, and in the larval episphere, with little indication of spatial staggering other than in the nervous system^14,20,26,27,40^. Scaphopod Hox transcripts are primarily present in the foot (*Hox2, Hox4, Hox5, Lox5, Lox4*, *Post1*), the mantle (*Hox1*), and in the central nervous system, with a near-to-staggered expression only in the mid-trochophore stage^37^. Cephalopods express some of these genes in the arm crown (*Hox1*, *Hox3*, *Hox4*, *Lox5*, *Lox4*, *Hox7*, *Post2*, *Post1*), the funnel tube (*Hox3*, *Lox5*, *Lox4*), and in various ganglia as well as in other clade-specific structures^22,29^. For bivalves, rudimentary expression data from *Hox1*, *Hox4*, *Lox5*, and *Post2* are available for the gastrula stage of a scallop only, wherein the authors interpreted their results as evidence of staggered expression^35^. In order to fill this gap in knowledge and to contribute to the question as to what degree Hox genes might contribute to bivalve-specific features, we here provide the first comprehensive dataset of tempo-spatial expression of Hox and ParaHox genes in the invasive freshwater mussel *Dreissena rostriformis*, a bivalve with an ancestral life cycle that involves indirect development via a trochophore and a veliger larva.

## Materials and methods

### Animal collection and cultures

Sexually mature individuals of *Dreissena rostriformis* were collected in the Danube river in Vienna, Austria (N 48° 14′ 45.812″, O 16° 23′ 38.145″). Collection took place between April and September 2017 and 2018, respectively. Adults were gathered from underneath stones and transferred to the laboratory. Here, the animals were washed, cleaned, and maintained in aquaria with filtered river water (FRW) at 19 °C.

Spawning of animals was induced by exposing 10–20 sexually mature specimens for 15 min to a 10^−3^ M solution of serotonin (Sigma-Aldrich, Darmstadt, Germany), followed by one wash and subsequent maintenance in FRW. After approximately 30 min, up to 50% of the treated specimens started to spawn. Oocytes were collected from the water column, inseminated, and cultured in 50 ml glass beakers at 23 °C. After fertilization, water was changed every half an hour for the first three hours and then every six hours to remove excess sperm and to avoid bacterial or fungal infection.

### RNA extraction and fixation of developmental stages

Several hundred individuals of each, cleavage, trochophore, and various veliger stages were stored in RNALater (Lifetechnologies, Vienna, Austria) at − 20 °C. RNA was extracted with an RNeasy Mini Kit (Qiagen, Hilden, Germany) according to the manufacturer’s instructions and was stored at − 80 °C.

Embryos and larvae from three developmental stages (gastrula, trochophore, and veliger) were fixed in 4% paraformaldehyde (PFA) in 0.1 M phosphate buffered saline (PBS) for 1 h at room temperature (23 °C). Veliger larvae aged 24 h post fertilization (hpf) or older were relaxed prior to fixation by adding cocaine crystals (Sigma-Aldrich, Darmstadt, Germany) to the FRW. All larvae were subsequently washed in 0.1 M PBS, transferred stepwise to 100% methanol, and stored at -20 °C.

### Transcriptome assembly and expression profiling

**Figure 1:**
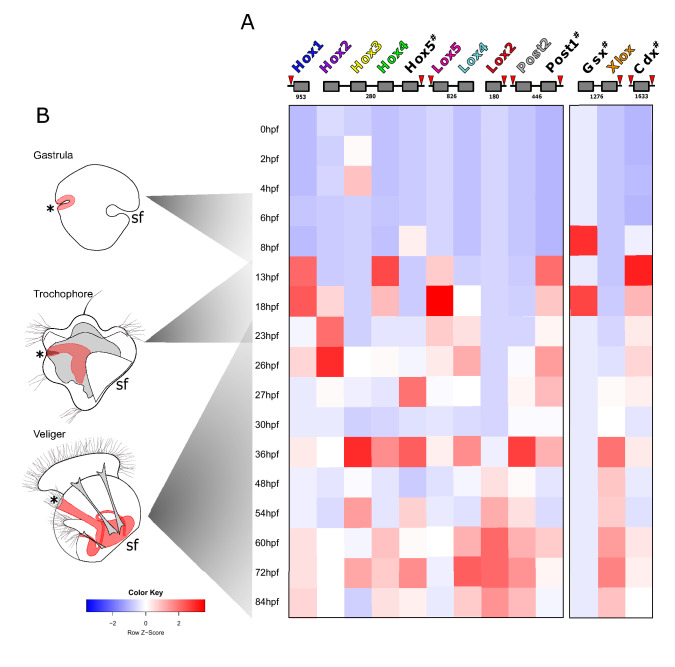
Relative quantitative analysis of Hox and ParaHox gene transcripts during development of *Dreissena rostriformis*. (**A**) Localization of Hox genes on scaffolds is shown in the upper part. Identification numbers of scaffolds where genes were found and location of non-Hox genes identified within the cluster are indicated by red arrowheads. Hashtags mark genes for which no expression data by in situ hybridization could be produced. (**B**) Heat map shows relative normalized expression levels for each gene (Z-score). Genes expressed above the normalization threshold are depicted in graded shades of red, those below the threshold in shades of blue. Time after fertilization and corresponding developmental stages at 23 °C are to the left. Germ layers and major derivatives in animal schemes are depicted in grey (mesoderm), red (endoderm), and white (ectoderm), respectively. Asterisks mark the blastopore/mouth, sf indicates the shell field.

RNA-seq transcription data collected from developmental stages of interest were assembled previously^41^. Gene expression levels of the 17 libraries were quantified with Kallisto (transcripts per kilobase million, TPM)^42^. Heatmaps showing relative quantitative expression of genes were plotted in R software with the heatmap.2 function from the gplots R package^43^ and columns were normalized by z-score (Fig. 1, Supplementary Table S1).

### Sequence alignment and phylogenetic analysis

Amino acid sequences of Hox orthologs were retrieved from published NCBI sequences of other molluscan, lophotrochozoan, and bilaterian gene orthologs. *Dreissena* Hox candidates were found in the previously assembled transcriptome by blasting against confirmed ortholog sequences. Gene names and GenBank accession numbers used for phylogenetic reconstruction are available in Supplementary Table S1. *Dreissena* Hox and ParaHox ortholog candidates were confirmed by searching for characteristic motifs in each gene from the quagga mussel transcriptome as previously described^33,41^. Amino acid sequences of candidate *Dreissena* Hox genes and metazoan orthologs were aligned using MAFFT v7.123b^44^. The trimmed multiple sequence alignment used for the phylogenetic analysis was obtained with TrimAl v1.2 with the following parameters: -cons 20 -st 0.8 -gt 0.7—w 1^45^. Model selection with Prottest3 v3.4.2^46^ through the Akaike Information Criterion (AIC) determined the Jones-Taylor-Thornton (JTT) model of amino-acid substitution as most appropriate for the alignment and thus was selected for the phylogenetic analysis. The maximum likelihood phylogenetic analysis was carried out with RAxML v8.2.X^47^ with gamma-distributed rates and 1,000 non-parametric bootstrap replicates. Bayesian analysis was performed using MrBayes v3.2.6^48^ with 25% of sampled trees discarded as burn-in, six rate categories for the gamma distribution, and 30,000,000 generations. The Bayesian phylogenetic run output was analyzed with the R package RWTY^49^. Likelihoods for the model parameters and resulting tree topologies were highlighted as a function of the number of sampled generations during the phylogenetic inference after burn-in of 25% (Supplementary Data 1). The phylogenetic trees were manually rooted using FigTree v1.4.4^50^.

### Gene cloning

Roche first-strand cDNA Synthesis Kit for rt-PCR (Roche Diagnostics, Mannheim, Germany) was used for cDNA synthesis from a pooled RNA sample of several larval stages (i.e., gastrula, trochophore and veliger). Identified Hox and ParaHox orthologs were used to design gene-specific primers. PCR amplified fragments were later separated by gel electrophoresis and purified with a QIAquick Gel Extraction Kit (Qiagen, Hilden, Germany). The products were cloned into pGEM-T Easy Vectors (Promega, Mannheim, Germany) and used to transform *E. coli* cells. Colonies were grown overnight, plasmids were later purified with a QIAprep Spin MiniprepKit (Qiagen) and sequenced for verification of insert orientation. For more descriptions of the whole experimental procedure see also^36,37,51–53^. Forward and reverse primers for each gene sequence can be found in Supplementary Table S2.

### Probe synthesis and whole mount in situ hybridization

Plasmid inserts were amplified by PCR amplification using M13 primers as described previously^36,52^. Antisense riboprobes were synthesized using digoxigenin-UTP (DIG RNA Labeling Kit, Roche Diagnostics) and SP6/ T7 polymerase (Roche Diagnostics).

For whole mount in situ hybridization, animals stored in 100% methanol were rehydrated in 0.1 M PBS and decalcified with PPE (20% PFA + 10 × PBS + 0.5 M EGTA at pH 8 + diethylpyrocarbonate; DEPC-treated H_2_O). Afterwards, samples were treated with proteinase-K at 37 °C for 10 min (10 µg/ml in PTw: 1 × PBS + 0.1% Tween-20). Samples were washed in PTw and post-fixed for 45 min in 4% PFA. Subsequently, samples were washed and transferred to hybridization buffer (50% formamide, 5 × SSC, 50 μg/ml heparin, 500 μg/ml yeast tRNA, 0.1% tween-20, pH 6.0) for 8–10 h at 56–60 °C. Probe hybridization was performed at the same temperature with probe concentrations ranging between 1 and 2 ng/µL for 30–48 h. Washing steps after hybridization were performed in wash buffer (75% hybridization buffer + 25% of SSC (3 M NaCl + 0.3 M saline-sodium citrate; SSC buffer + DEPC H_2_O)) three times for 10 min each, then twice in 50% hybridization buffer + 50% SSC for 10 min each, and finally three times in 25% hybridization buffer + 75% SSC for 7 min (once) and 1xSSC + 0.1%Tween-20 for 5 min each. Next, three washes in 0.1 M MAB (maleic acid buffer) were performed. A digoxigenin(DIG)-labeled alkaline phosphatase (AP)-antibody raised in sheep (Roche Diagnostics) was used at a dilution of 1:5,000 in blocking solution (10 × blocking reagent, Roche Diagnostics, + 0.1 M MAB) at 4 °C overnight. Samples were then washed in PTw three times for 20 min each and twice for 10 min each. Development of the color reaction was done in a NBT/BCIP solution (Roche Diagnostics) diluted in 1 × alkaline phosphatase buffer (0.5 M NaCl + 0.5 M Tris at pH 9.5 + 50 mM MgCl_2_ + 0.1%Tween-20 + DEPC H_2_O) at 4 °C with periodic buffer replacement until signal was detected. Color reactions were stopped by washing five times for 10 min each in PTw.

### Image collection and figure processing

Larvae were mounted in 70% glycerol and examined and documented with an Olympus BX53 Microscope (Olympus, Hamburg, Germany). Light micrographs were processed in Inkscape (version 0.92.4–4) for contrast and brightness. All figures and graphical representations were prepared in Inkscape (version 0.92.4–4).

## Results

### Phylogenetic analysis of gene orthologs

Candidate orthologs of all ten Hox genes (*Hox7* appears to have been lost as in *Crassostrea gigas*) and three ParaHox genes were identified from the quagga mussel transcriptome. Multiple sequence alignment of the putative Hox proteins of *Dreissena rostriformis* and other bilaterians shows high sequence conservation within the homeodomain regions (Supplementary Data 2). Tree topologies obtained from Bayesian inference and maximum likelihood methods are highly consistent and reveal similar clusters among corresponding ortholog groups. All *Dreissena* gene candidates cluster with either a mollusk or another lophotrochozoan ortholog sequence (Supplementary Table S3, Supplementary Data 3, 4). *Dro-Hox1-5*, *Dro-Lox2, Dro-Lox4, Dro-Lox5*, *Dro-Post1*, and *Dro-Post2,* together with the ParaHox sequences *Dro-Gsx, Dro-Xlox*, and *Dro-Cdx*, were successfully cloned and the respective riboprobes were synthesized. All probes except those for *Dro-Hox5*, *Dro-Post1*, *Dro-Cdx*, and *Dro-Gsx* yielded results by in situ hybridization. The negative results of the latter were most likely due to low expression levels of these genes in the targeted developmental time points (Supplementary Table S1, Supplementary Data 5).

### Hox and ParaHox gene arrangement

*Dreissena rostriformis* Hox and ParaHox genes are distributed along several scaffolds of the genome, which may either reflect the fragmented state of the assembly or disruption of the respective clusters (Supplementary Table S4, Supplementary Data 6). The Hox genes were distributed over five scaffolds, while two of the three ParaHox genes (*Gsx*, *Xlox*) were found on a single scaffold. Several non-Hox genes were found between members of the Hox cluster (between *Xlo*x and *Cdx*, *Hox5* and *Lox5*, *Lox2* and *Post2*), demonstrating insertions all along the cluster (Fig. 1a, Supplementary Table S4, Supplementary Data 6). This indicates a somewhat disorganized (albeit not broken) Hox cluster^12^.

### Hox and ParaHox gene expression in *Dreissena rostriformis*

**Figure 2:**
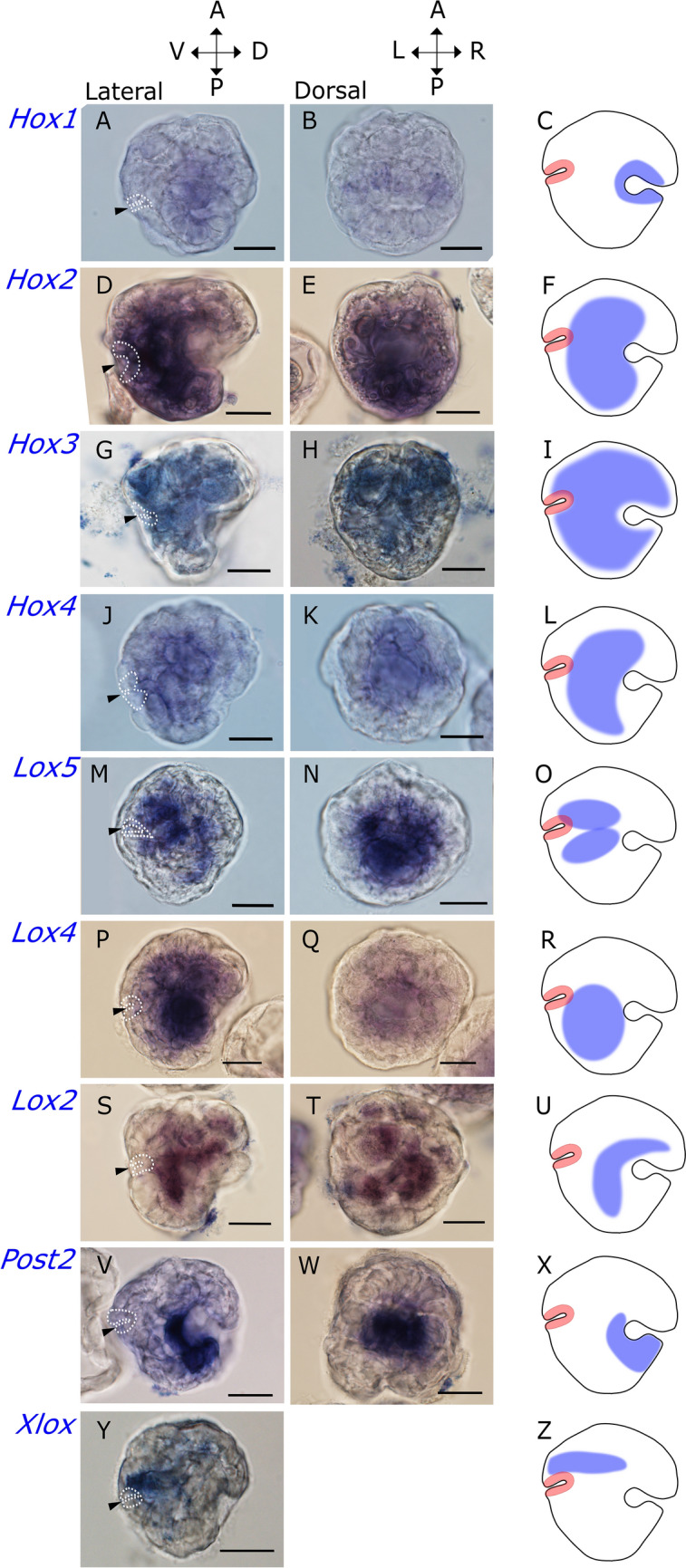
Hox gene expression in the late gastrula stage of *Dreissena rostriformis*. Graphical representation of the corresponding expression domains is to the right. Hox genes are not expressed in a staggered fashion. In the schemes, the future ectoderm is marked in white and the developing endoderm in red. Sites of gene expression are labeled blue and largely correspond to domains of future mesoderm. In the micrographs, the developing endoderm (site of gastrulation) is marked by dotted lines. (**A**–**C**) *Hox1* is expressed at the anterior margin of the shell field. (**D**–**F**) *Hox2* is expressed in the median region of the developing mesoderm. (**G**–**I**) *Hox3* is present in the antero-ventral region around the mouth. (**J**–**L**) *Hox4* is found in separate regions in the developing mesoderm. (**M**–**O**) *Lox5* is constrained to the ventro-median mesoderm. (**P**–**R**) *Lox4* is expressed in tissue underlying the posterior margin of the shell field. (**S**–**U**) *Lox2* is expressed in the mesoderm that extends towards the posterior region. (**V**–**X**) *Post2* is found in the posterior margin of the shell field. (**Y**,**Z**) *Xlox* is expressed in a group of cells in an anterior mesodermal domain. Arrowheads mark the blastopore. a, anterior; p, posterior; v, ventral; d, dorsal; l, Left; r, right. Scale bars equal 20 µm.

**Figure 3:**
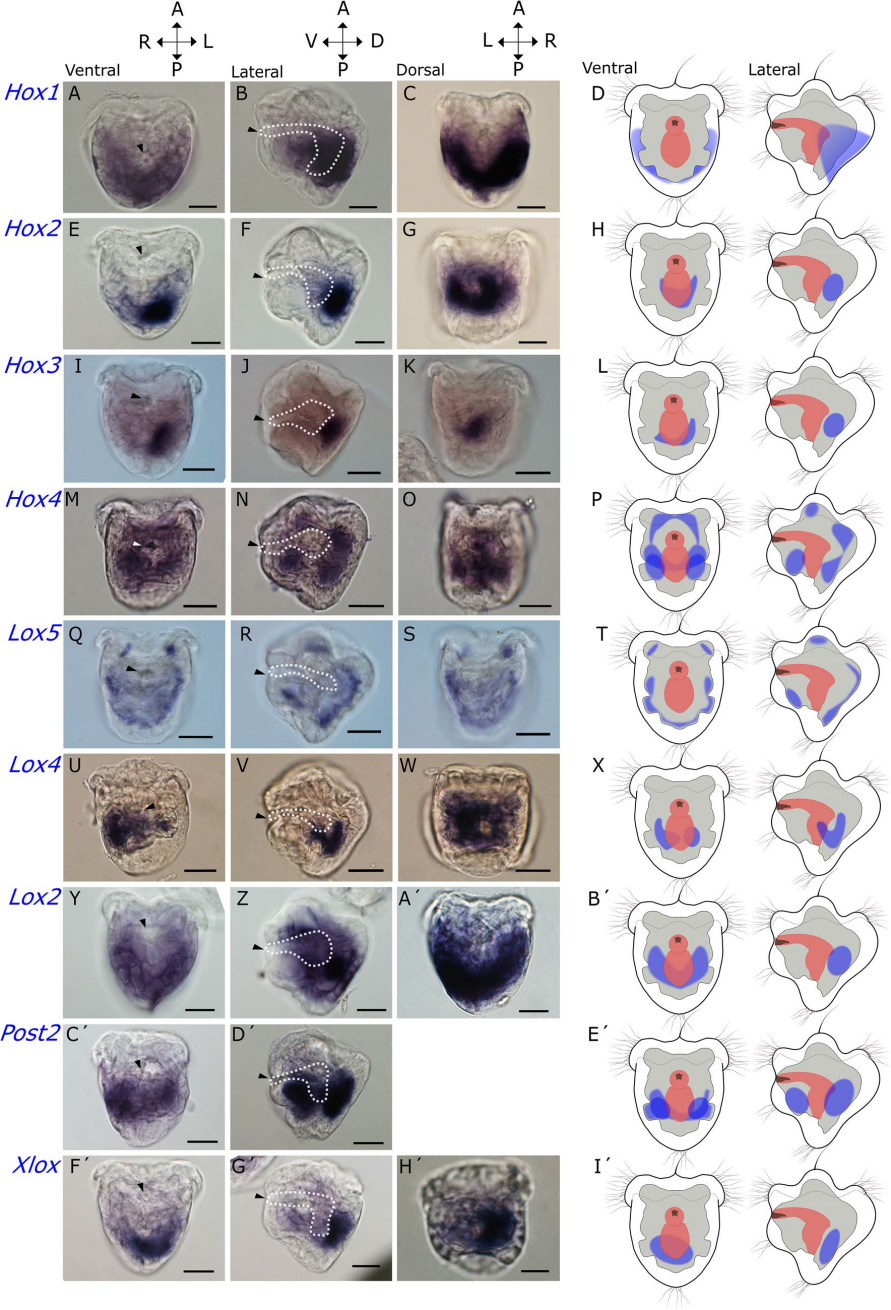
Hox gene expression in the trochophore stage of *Dreissena rostriformis*. Graphical representation of the corresponding expression domains is to the right with outline of the developing digestive tract in red, ectodermal domains in white, mesoderm in grey, and sites of gene expression in blue. In the micrographs, dotted lines mark the developing digestive tract. Hox genes are mostly co-expressed in regions of the developing mesoderm in a non-staggered fashion. (**A**–**D**) *Hox1* is expressed in all cells of the shell field, i.e. in domains dorso-lateral of the developing digestive tract. (**E**–**H**) *Hox2* is expressed in the mesodermal layer that underlies the shell field. (**I**–**L**) *Hox3* is expressed in the mesoderm that underlies the shell. (**M**–**P**) *Hox4* is expressed in separated cell masses belonging to the developing mesoderm. (**Q**–**T**) *Lox5* is expressed in individual mesodermal cell clusters throughout the larval body. (**U**–**X**) *Lox4* is expressed in the dorsal mesoderm that underlies the shell field. (**Y**–**B**′): *Lox2* is expressed in the dorsal mesoderm below the shell field and surrounds parts of the developing digestive tract. (**C′**–**E′**) *Post2* is found on the postero-ventral side. (**F**′–**I**′) *Xlox* is expressed in cells that lie adjacent to the developing hindgut. Arrowheads mark the mouth opening. a, anterior; p, posterior; v, ventral; d, dorsal; l, left; r, right. Scale bars equal 20 µm.

First detection of Hox and ParaHox gene expression by in situ hybridization started in the late gastrula stage (i.e. after 10hpf), in which *Dreissena* is heavily ciliated yet lacks a distinct mesodermal layer. The large invagination of the shell field marks the future dorsal side, and the smaller invagination of the blastopore lies on the opposite, ventral side (Fig. 2). Although this time point was not directly sampled for quantitative analysis, data from 8 and 13hpf old individuals suggest that significant transcription rates start between these stages. Both Hox and ParaHox gene expression is maintained throughout the trochophore stage (i.e. around 13-16hpf) in which larvae become slightly elongated, the shell field has already evaginated, and the prototroch is distinct (Fig. 3). Hox and ParaHox gene expression was not detected in veliger stages.

#### *Hox 1*

Expression of *Dro-Hox1* is restricted to the shell field in the late gastrula and in the trochophore stage (Figs. 2a–c, 3a–d). Expression of *Hox1* is initially found at the anterior margin of the shell field in gastrula stage. In the trochophore larva the expression domain expands to all cells of the shell field.

#### *Hox 2*

*Dro-Hox2* shows strong expression in the median region of the developing mesoderm of the gastrula and is located towards the posterior half of the embryo (Fig. 2d–f). Subsequently, mesodermal expression becomes confined to the median region of the trochophore that corresponds to the mesodermal layer underlying the shell field (Fig. 3e–h).

#### *Hox 3*

*Dro-Hox3* is widely expressed in the mesoderm of the gastrula. Its expression domain partially overlaps with *Hox2*, although *Hox3* transcripts are distributed more widely all across the larval body (Fig. 2g–i). The unspecific stain around the periphery of the gastrula constitutes non-larval material. In the trochophore, *Hox3* expression overlaps with most genes except *Hox1*, *Post2*, and *Xlox*. Expression is confined to a mesodermal domain that underlies the shell field (Fig. 3i–l).

#### *Hox 4*

In the gastrula stage, low levels of *Dro-Hox4* expression are evident in the developing mesoderm, with expression overlapping with *Dro-Hox2* and *Dro-Hox3* expression domains. In the late trochophore, *Hox4* is expressed in separated cell clusters that belong to the developing mesoderm and are distributed all over the larval body (Figs. 2j–l, 3m–p).

#### *Lox 5*

In the gastrula stage, the expression domain of *Lox5* partially overlaps with *Dro-Hox2, Dro-Hox3*, and *Dro-Hox4*. Expression at this stage is restricted to the presumptive ventro-median mesoderm (Fig. 2s–u). In the late trochophore stage, gene expression occurs in individual mesodermal cell clusters throughout the larval body (Fig. 3q–t).

#### *Lox 4*

In the gastrula, expression of *Dro-Lox4* is located in mesodermal tissue that underlies the posterior margin of the shell field (Fig. 2p–r). In the trochophore stage, *Dro-Lox4* is expressed in the dorsal mesoderm that underlies the shell field (Fig. 3u–x).

#### *Lox 2*

*Dro-Lox2* shows a clear shift of expression between developmental stages. In the gastrula stage, the expression is in median mesodermal tissue that extends towards the posterior region of the embryo. In the trochophore, transcripts are located in the dorsal mesoderm below the shell field. The pattern overlaps with *Dro-Hox2*, *Dro-Hox3*, and, to some extent, with *Dro-Lox4* (Figs. 2m–o, 3y–b′).

#### *Post 2*

*Dro-Post2* expression is located in cells of the posterior margin of the shell field in the gastrula, opposite to *Dro-Hox1* (Fig. 2v–x). In the trochophore stage, transcripts are located more posteriorly on the ventral side of the larva, where expression co-localizes with the foot anlage (Fig. 3c′–e′).

#### *Xlox*

*Dro-Xlox* is first detected in the gastrula stage, in a group of cells corresponding to the anterior region of the developing digestive tract, i.e. in an endodermal domain, and in parts of the emerging anterior mesoderm (Fig. 2y, z). In the trochophore, *Dro-Xlox*-expressing cells lie adjacent to the developing hindgut (Fig. 3f′–i′).

## Discussion

### Non-staggered Hox gene expression in *Dreissena*

Hox genes in bilaterians are predominantly expressed along the anterior-posterior axis. They determine regionalization of morphological structures across the animal body. Since this staggered spatial expression is present in most clades, it is considered ancestral for Bilateria^9–12,54,55^. In bivalves, similar spatially staggered expression of *Hox1*, *Hox4*, *Lox5*, and *Post2* were proposed for a scallop, *Mizuhopecten yessoensis*. However, only the gastrula stage was investigated in this bivalve and data are therefore largely incomplete. Transcriptomic data on this scallop seem to suggest a subcluster-level of temporal staggered expression whereby the first genes of each subcluster are expressed simultaneously, followed by the sequential expression of each subsequent gene within each subcluster^35^. By contrast, stage-specific quantitative transcriptome data for another bivalve, *Crassostrea gigas*, appears to more closely reflect the results from *Dreissena* insofar as no evidence of sequential or collinear activation of Hox genes was found^28,56^.

In *Dreissena*, the relative expression domains of a few genes (*Hox1, Post2, Lox4*) might be interpreted as “partially spatially staggered” in the gastrula stage. *Dro-Lox4* maintains its pattern of expression in the dorso-posterior half of the gastrula and the trochophore, while *Dro-Hox1* is expressed anteriorly in the dorsal region of the gastrula. However, during the transition to the trochophore stage, *Dro-Hox1* expands throughout the entire shell field. Such a *Hox1* expression domain has also been found in gastropods, scaphopods, and the scallop, and might constitute an autapomorphy of Conchifera^26,35,37,40^. All other *Dreissena* Hox genes display highly dynamic spatial expression profiles with changes in expression domains frequently occurring between the gastrula and trochophore stages. As such, *Dro-Post2* expression is initially restricted to the posterior margin of the presumptive shell field, in close proximity to *Dro-Hox1*, but later extends ventro-posteriorly in the trochophore. This non-staggered expression of *Dro-Hox1, Dro-Hox2, Dro-Hox3, Dro-Hox4, Dro-Lox2, Dro-Lox5, Dro-Post2*, and *Dro-Xlox* is also reflected by their non-collinear but rather synchronous temporal activation (Fig. 1, Supplementary Data 5).

### Cooption of Hox genes in mollusks

**Figure 4:**
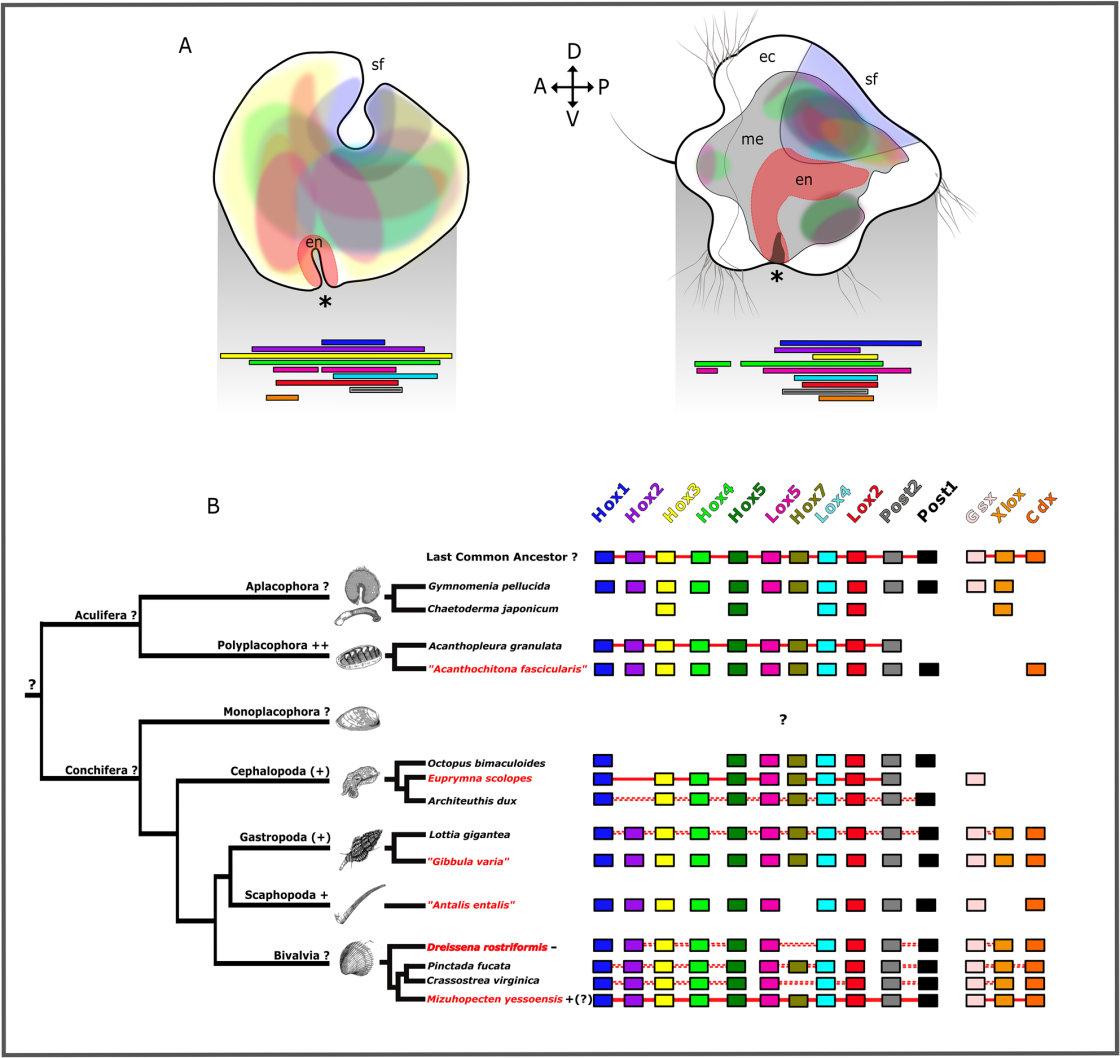
Schematic representation of Hox gene expression in *Dreissena rostriformis*. Summary of Hox expression patterns together with the localization of Hox genes in the genome of selected mollusk species. (**A**) Hox genes in the late gastrula (left) and the late trochophore larva (right) do not follow a temporally or spatially staggered mode of expression. (**B**) Hox gene arrangement in mollusks. Phylogenetic tree adapted from^1,66^, animal schemes modified after^67^. Data from^16,18,19,35,39,57,68^. Straight red lines indicate absence of coding sequences between individual Hox genes, dotted red lines indicate presence of non-Hox genes between Hox genes, blank space between Hox genes means that presence or absence of non-Hox genes between Hox genes remains unknown. Species for which gene expression data by in situ hybridization are available are highlighted in red. ++ marks lineages with clear spatially staggered Hox expression, + indicates prominent hints of spatial Hox expression, ( +) symbolizes weak hints of spatial expression, - indicates absence of staggered expression, ? indicates that no clear statement concerning staggered Hox expression can be made. Note that the situation in *Mizuhopecten yessoensis* is somewhat undecided since only few Hox genes were investigated in the gastrula stage only. Quotation marks indicate that species identity is not fully settled (*G. varia*, *A. entalis*) or that genomic and gene expression data for the identical species have been published under different species names (*A. fascicularis*, where gene expression data were erroneously assigned to *A. crinita*). a, anterior; d, dorsal; ec, ectoderm; en, endoderm; me, mesoderm; p, posterior; sf, shell field; v, ventral; asterisk, blastopore/mouth opening.

Conchiferan Hox gene expression is often located in specific developing morphological structures and does not strictly follow an anterior–posterior gradient, although rudiments of an ancestral staggered mode of expression are recognized to varying degrees in individual lineages and developmental stages^16,18,26,36,37,53^ (Fig. 4b). *Hox2, Hox3, Hox4*, *Lox5*, *Lox4*, and *Lox2* show an overlapping expression in the developing mesoderm in the gastrula stage in *Dreissena* (Figs. 2, 4a). Subsequently, the expression domains still overlap, but are more confined to dorsal parts of the mesoderm (Figs. 3, 4a). In other conchiferans, Hox genes show more distinct spatial staggering along the anterior–posterior axis. In pre-torsional gastropod larvae, *Hox2, Hox3, Hox4, Hox5*, and *Hox7* expression domains appear somewhat staggered in the developing nervous system, while *Hox1, Lox5, Lox4, Lox2, Post2*, and *Post1* do not. Instead, these genes are expressed in distinct morphological features such as the shell field, foot, prototroch, and the larval apical organ^14,20,26,37,38,40^. A similar situation is found in cephalopods, where *Hox1, Hox3, Hox5, Lox4*, and *Post2* expression is located in the arm crown of the bobtail squid *Euprymna scolopes* in a somewhat staggered manner, but are also active in the pedal ganglia, the palliovisceral ganglia, and the light organ. *Hox3, Hox5, Lox4*, and *Lox5* are additionally expressed in the developing funnel^21,22^. Trochophore larvae of the scaphopod *Antalis entalis* likewise show spatially staggered expression of seven out of nine *Hox* genes, with *Hox2*, *Hox4*, *Hox5, Lox5, Lox4*, and *Post1* being expressed in the anlagen of the ganglia, the mantle margin, and the foot^37^. Taken together, it appears that Hox genes have been recruited independently into novel expression domains in various conchiferan lineages and have most likely also acquired novel roles at the cost of their original function in anterior–posterior axis specification.

Interestingly, and strikingly different from any conchiferan investigated to date, the aculiferan polyplacophoran *Acanthochitona fascicularis* shows almost textbook-like spatial (but not temporal) Hox gene expression along the anterior–posterior axis in the trochophore stage^30,31,38^. Here, Hox genes are not confined to distinct morphological features but instead to well-defined axial territories, thus probably resembling the conserved bilaterian condition^11^. Moreover, the genome of the polyplacophoran *Acanthopleura granulata* shows evidence of an intact cluster of the 11 Hox genes^57^. This supports the assumption that the last common ancestor of at least Aculifera likewise might have showed staggered and possibly collinear expression of Hox genes, although a final argument cannot be made without data from the aplacophoran clades (Fig. 4b). The deviation from this ancestral condition and deployment into novel roles in the conchiferan lineages might have facilitated the evolution of the immense diversity of body plans in the Conchifera^39^.

### Comparative aspects of ParaHox gene expression

ParaHox genes are a set of genes which originated from a duplication of the ancestral metazoan Proto-Hox cluster^58^. They comprise the *Cdx, Xlox*, and *Gsx* genes, which are commonly expressed in a staggered fashion in the hindgut, midgut, and foregut, respectively. Thus, it is assumed that ParaHox genes have an ancestral function in digestive tract patterning and regionalization^58,59^. This is supported by staggered expression of these genes along the developing gut in the polychaete annelids *Platynereis dumerilii*, *Alitta virens*, and *Capitella teleta*^60–62^. Data from the polyplacophoran *Acanthochitona* support this notion, with *Cdx* being expressed in the region of the developing hindgut, but data on other ParaHox genes are still lacking^31^. Once again, the conchiferans deviate from this condition. Although gastropod ParaHox genes appear spatially staggered expressed in both the gut and developing ganglia^27^, *Gsx* is expressed in the developing nervous system of scaphopods and cephalopods^36^. Interestingly, *Dro-Xlox* expression in *Dreissena* resembles more the condition of other bilaterians including deuterostomes, where it is expressed in the midgut, rather than the one of its conchiferan relatives^60–65^. Thus, in contrast to the Hox gene condition, our results suggest that in *Dreissena*, *Xlox* has retained its ancestral expression domain reminiscent of the last common bilaterian ancestor.

### Staggered Hox expression versus body plan regionalization in Mollusca

The Hox complement of Mollusca comprises 11 genes. These are expressed in a staggered fashion in polyplacophorans, a representative of the aculiferan lineage with a number of putative conserved ancestral traits such as a serially arranged dorso-ventral musculature, a nervous system with non-ganglionated longitudinal nerve cords, an unpronounced cephalic region, and an elongated foot that extends more than three quarters along the longitudinal body axis^30^. The non-regionalized anterior–posterior distribution of these morphological traits and the conserved mode of staggered Hox gene expression coincides with the situation in the polychaete annelid *Platynereis* which, despite significant differences with the polyplacophoran body plan such as segmentation and ganglionated nerve cords, likewise shows a largely evenly distributed set of organ systems along its longitudinal axis as well as staggered expression of Hox genes^54^. From this common scheme, conchiferan molluscs have decoupled Hox gene expression from their original position along the anterior–posterior axis in various ways, depending on the degree of body regionalization. As such, scaphopod larvae with a pronounced longitudinal axis with relatively little structural concentration have largely retained the staggered mode of Hox expression, while gastropod veligers and cephalopod embryos with a high degree of organ system concentration, particularly in the anterior region, only show hidden traits of staggered Hox expression^37^. This independent loss (to varying degrees) of staggered Hox gene expression in the scaphopod-gastropod-cephalopod lineages has been replaced by coopted expression of these genes in (sometimes lineage-specific) distinct morphological structures such as the shell, the foot including funnel, the tentacles, or the larval prototroch. The situation in the bivalve *Dreissena* differs from that of their conchiferan (and polyplacophoran) relatives insofar as Hox genes are detected by in situ hybridization only until the trochophore stage, a larval type with little structural complexity. By the time the complex and regionalized veliger larva starts to establish, Hox gene expression is lost. Thus, the widely overlapping, non-staggered expression domains in the *Dreissena* trochophore and the low expression level during veliger patterning indicate that these genes might, at best, play a minor role in axis patterning in this bivalve, similar to the gastropods and cephalopods.

In conclusion, the data currently available corroborate the high plasticity of Hox gene expression in Mollusca. The use of functional assays and gene manipulation tools such as RNAi or the CRISPR/Cas9 system could help elucidate the function of individual Hox genes and the molecular basis of the developmental and evolutionary pathways that have resulted in the manifold morphological novelties in conchiferan mollusks.

## Supplementary information

Supplementary Information
