# Supplementary material for: Non-collinear Hox gene expression in bivalves and the evolution of morphological novelties in mollusks
Source: Sci Rep. 2021 Feb 11;11:3575. doi: 10.1038/s41598-021-82122-6 (PMC7878502; doi:10.1038/s41598-021-82122-6)
Supplement: Supplementary file 1 — Supplementary Information [file 41598_2021_82122_MOESM1_ESM.pdf]

Title: Non-collinear Hox gene expression in bivalves and the evolution of morphological novelties in mollusks

Authors: David A. Salamanca-Díaz[1], Andrew D. Calcino[1], André L. de Oliveira[2], Andreas Wanninger[1,] *

[1]University of Vienna, Department of Evolutionary Biology, Unit for Integrative Zoology, Althanstraße 14, 1090 Vienna, Austria

[2]University of Vienna, Deparment of Functional and Evolutionary Ecology, Unit for Bio-Oceanography & Marine Biology, Althantraße 14, 1090 Vienna, Austria

* author for correspondence: Andreas Wanninger; email: andreas.wanninger@univie.ac.at

ORCID nr. Andreas Wanninger: 0000-0002-3266-5838

Supplementary Data 1. Examining Convergence of Bayesian Phylogenetic Analysis. A and B: Computed likelihoods for the selected model parameters. C and D: Topological distance for the resulting tree topologies from the focal tree. All above as function of the number of sampled generations during the phylogenetic analysis (after a burn-in of 25%). E: Cumulative change in split frequencies as a function of chain length. F: Split frequency comparisons showing convergence on topology, supporting values, and posterior probabilities for all clades.

Supplementary Data 2. Raw alignment of homeodomains and flanking regions of both directions without trimming of gene candidates and ortholog sequences. Columns are colored following the ClustalX color scheme based on a minimum percentage identity threshold (>30%). The alignment conservation annotation represented by the histograms is the quantitative numerical index that reflects the conservation of the amino acids' physico-chemical properties for each column of the alignment.

Supplementary Data 3. Phylogeny of *Dreissena rostriformis* Hox and ParaHox genes analysed in this study. Phylogenetic tree built from amino acid sequences of candidate orthologs produced with Bayesian inference**.** Branch support values are indicated on each major branch as posterior probability values. Scale bar in the lower left corner shows amino acid substitution rate per site. Red arrowheads mark sequences from *Dreissena rostriformis*.

Supplementary Data 4. Phylogeny of amino acid sequences of candidate orthologs inferred by maximum likelihood. Bootstrap support values are indicated on each major branch. Scale bar in the lower left corner shows amino acid substitution rate per site. Arrows point at candidate sequences from *Dreissena rostriformis*.

Supplementary Data 5. Relative expression levels (transcripts per kilobase million, TPM) of *Dreissena rostriformis* Hox genes. A: Histogram grouping individual expression levels of all detected genes. B-N: Individual quantities for each gene through developmental time. Raw values are found in Supplementary Table 1.

Supplementary Data 6. General Feature Format (GFF3) file providing information for exonic regions and coding region sequences (CDS) from all identified Hox, ParaHox, and non-Hox genes found on the respective scaffolds in the genome of *Dreissena rostriformis*.

Supplementary Table 1. Raw data of relative expression levels (transcripts per kilobase million, TPM) of *Dreissena rostriformis* Hox genes throughout development.

Supplementary Table 2. List of primers for each gene used for cloning. Forward and reverse primers used for amplifying each gene by PCR are indicated.

Supplementary Table 3. Orthologs listed by taxonomic classification, code names, and accession numbers used for the phylogenetic reconstructions in this study.

Supplementary Table 4. Scaffold characteristics of the genome assembly. Genes found in each scaffold and the corresponding sizes are indicated.

Supplementary Data 1

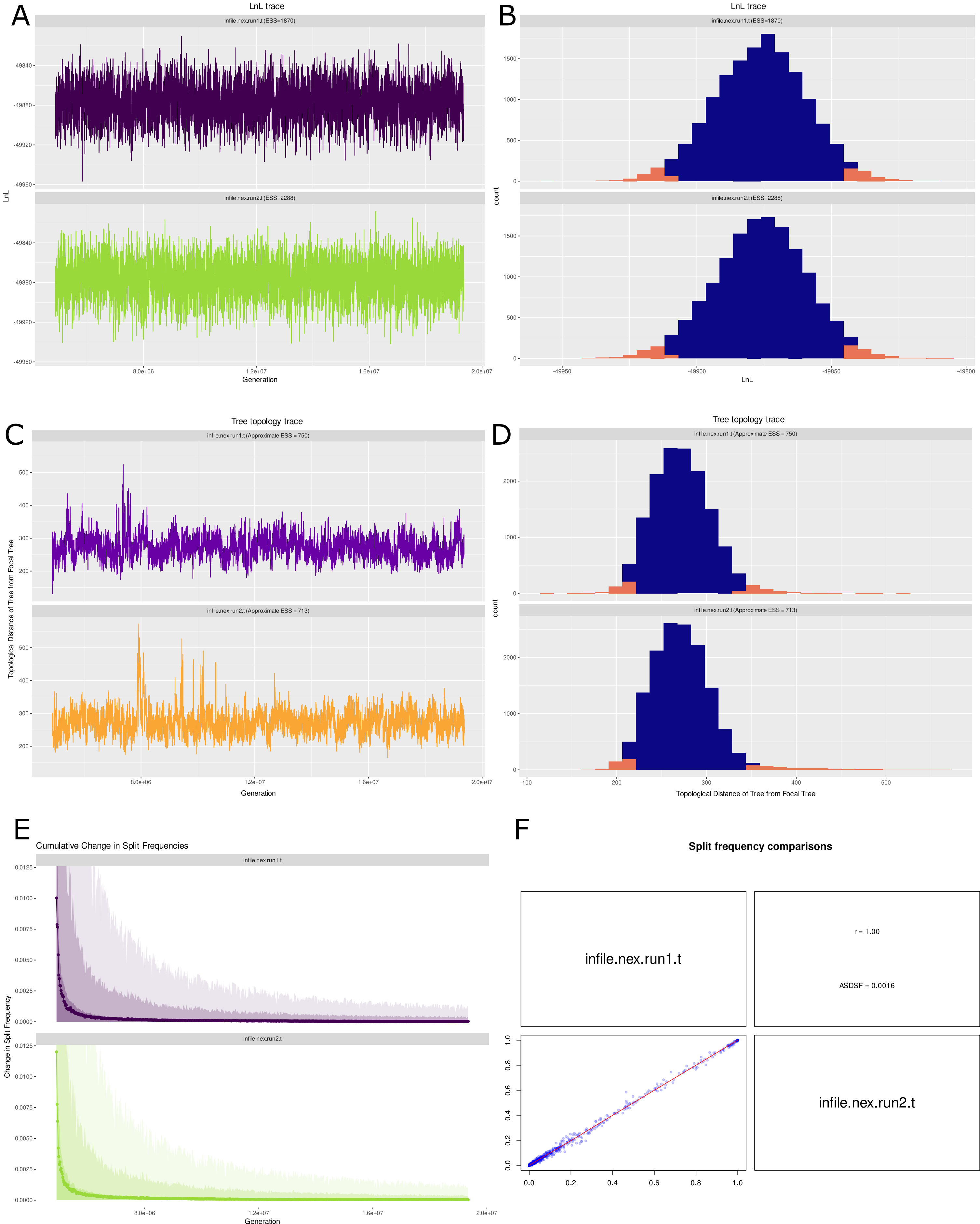

Supplementary Data 2

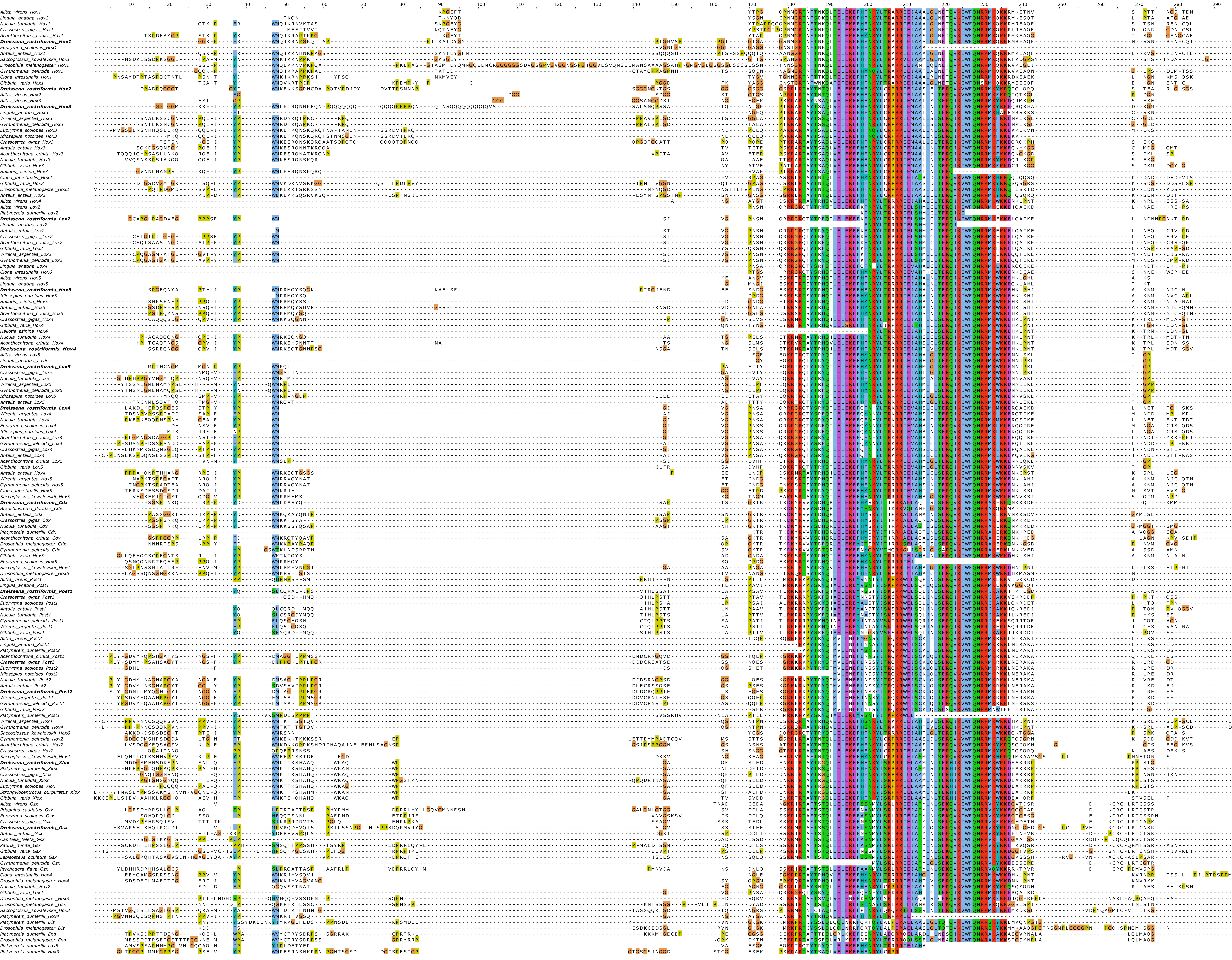

Supplementary Data 3

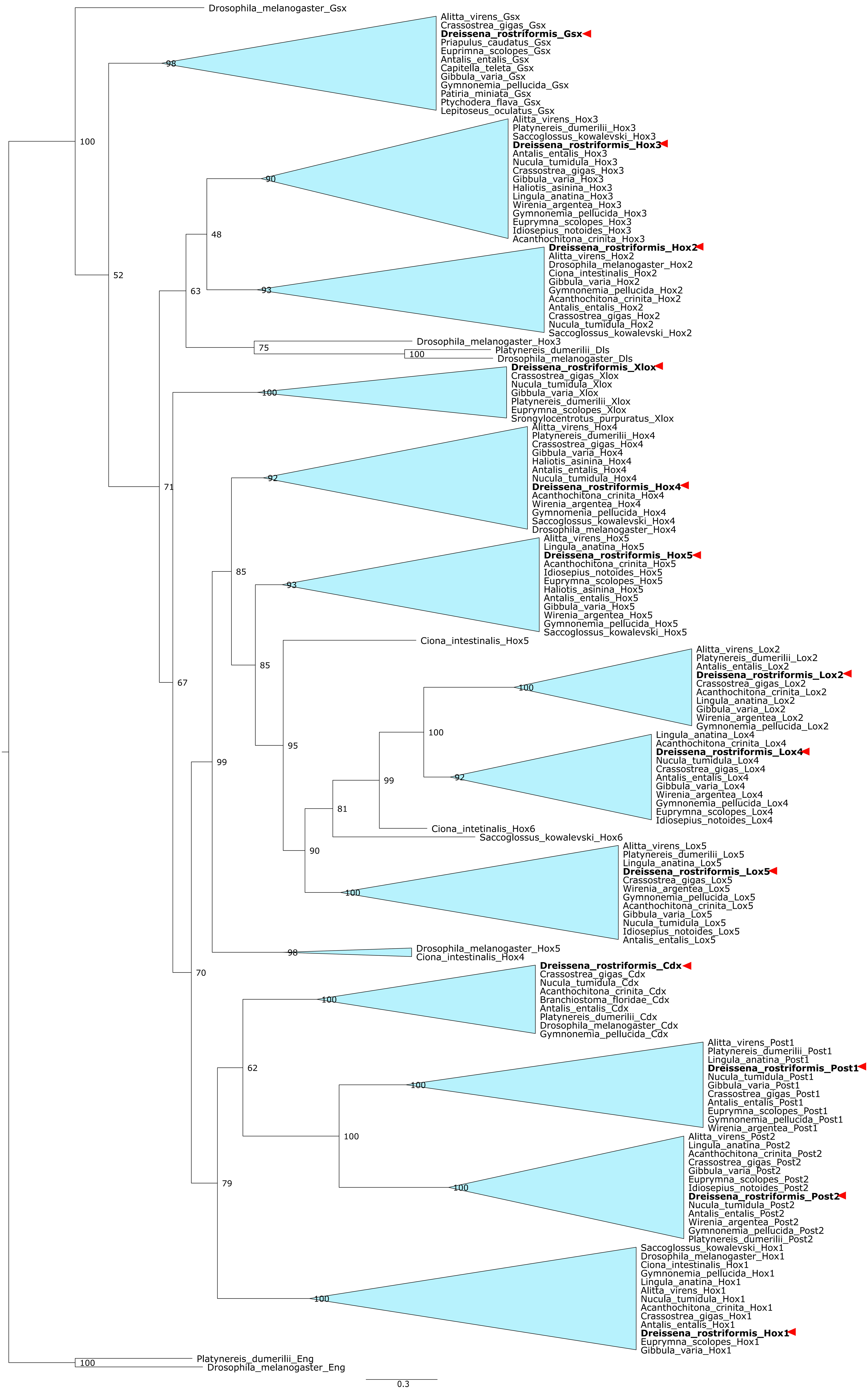

Supplementary Data 4

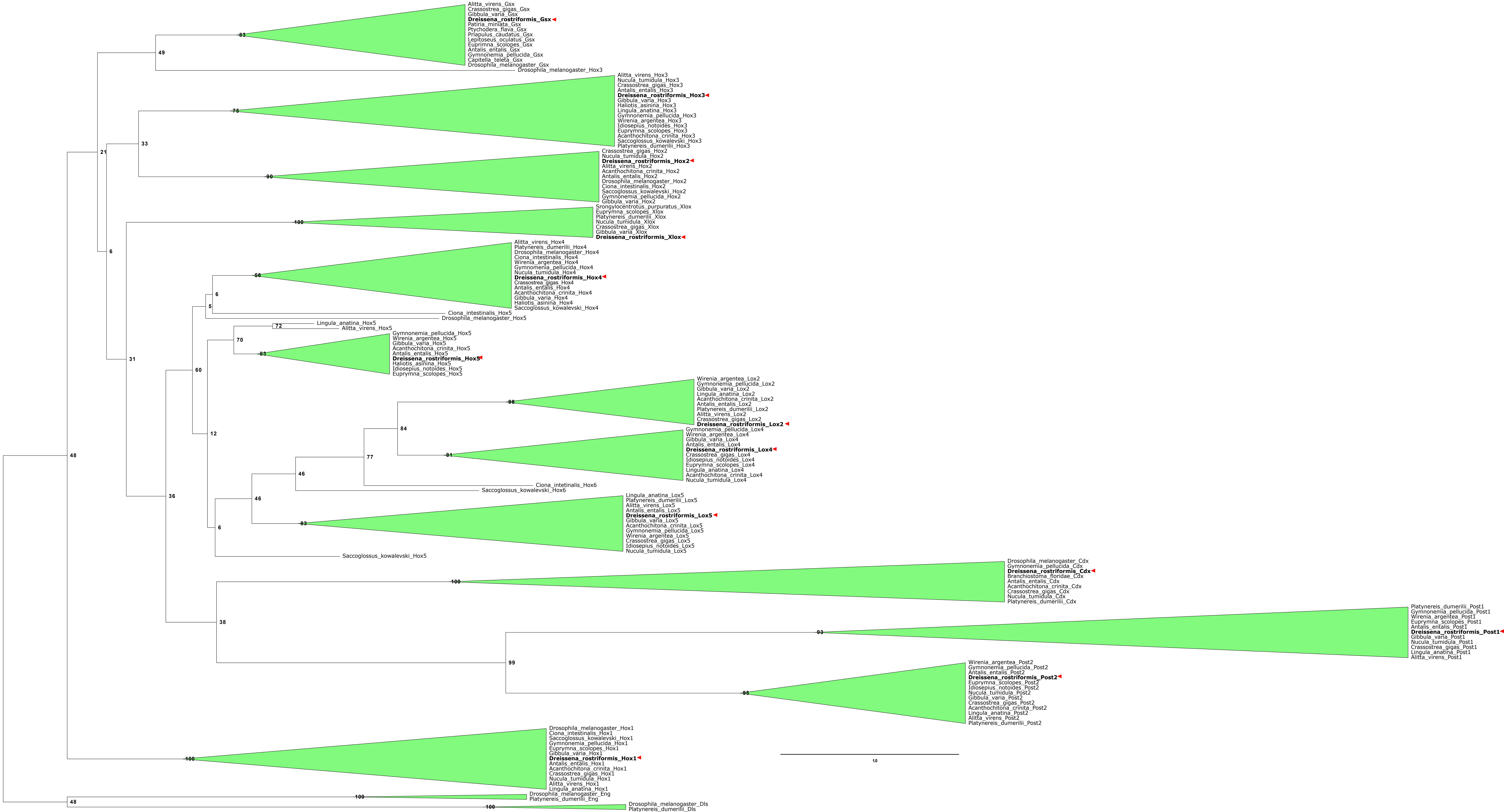

Supplementary Data 5

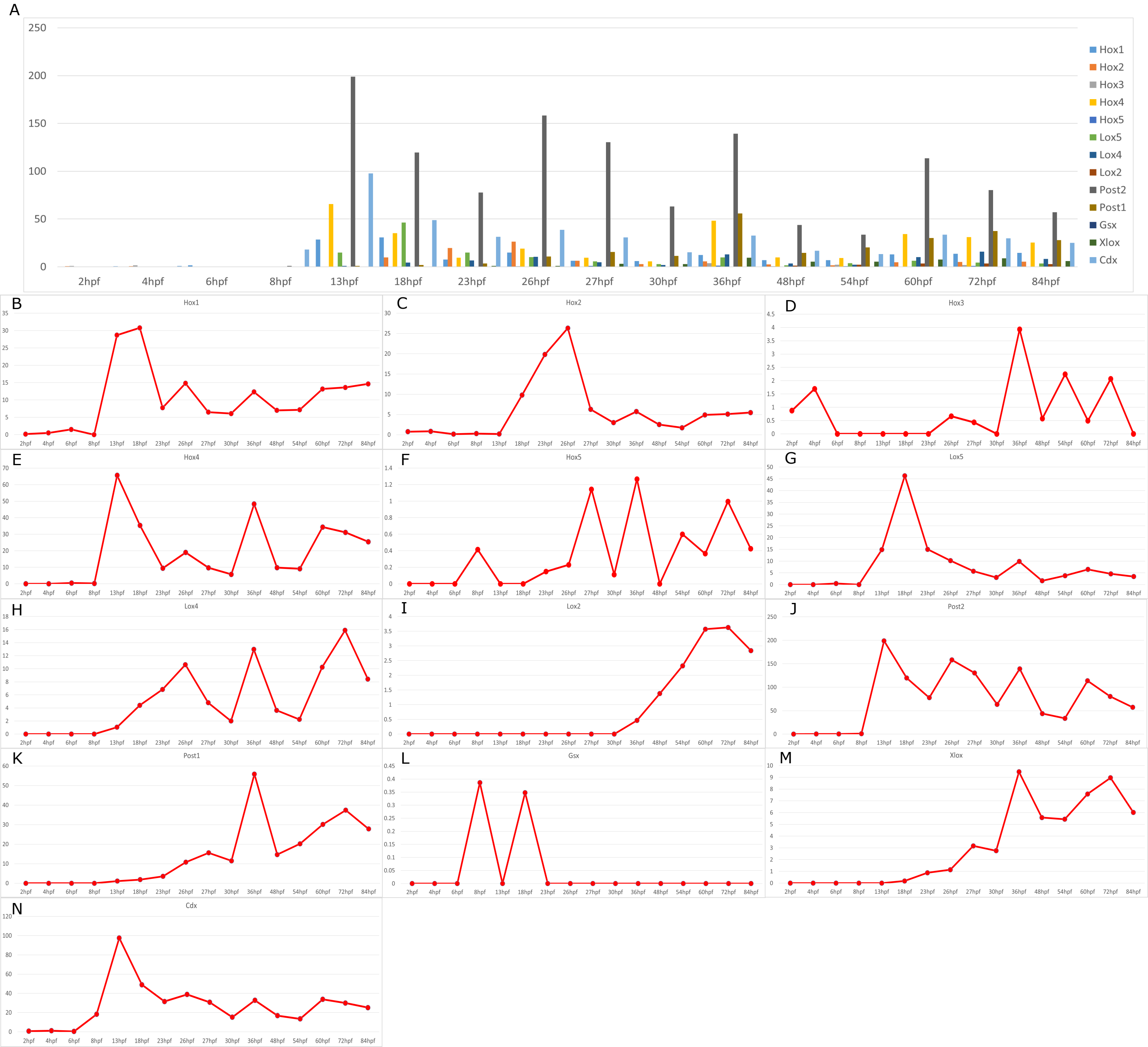

Supplementary Data 6

scaffold953	transdecoder	gene	124527	132034	.	+	.	ID=97299;Name=ORF
scaffold953	transdecoder	mRNA	124527	132034	.	+	.	ID=Gene.152836;Parent=97299;Name=ORF
scaffold953	transdecoder	exon	124527	124616	.	+	.	ID=Gene.152836:exon1;Parent=Gene.152836
scaffold953	transdecoder	CDS	124527	124616	.	+	0	ID=cds.Gene.152836;Parent=Gene.152836
scaffold953	transdecoder	exon	127742	127841	.	+	.	ID=Gene.152836:exon2;Parent=Gene.152836
scaffold953	transdecoder	CDS	127742	127841	.	+	0	ID=cds.Gene.152836;Parent=Gene.152836
scaffold953	transdecoder	exon	131808	132034	.	+	.	ID=Gene.152836:exon3;Parent=Gene.152836
scaffold953	transdecoder	CDS	131808	132034	.	+	2	ID=cds.Gene.152836;Parent=Gene.152836
scaffold953	transdecoder	gene	151405	173339	.	-	.	ID=97301;Name=ORF
scaffold953	transdecoder	mRNA	151405	173339	.	-	.	ID=Gene.152834;Parent=97301;Name=ORF
scaffold953	transdecoder	exon	173003	173339	.	-	.	ID=Gene.152834:exon1;Parent=Gene.152834
scaffold953	transdecoder	CDS	173003	173339	.	-	0	ID=cds.Gene.152834;Parent=Gene.152834
scaffold953	transdecoder	exon	151775	152120	.	-	.	ID=Gene.152834:exon2;Parent=Gene.152834
scaffold953	transdecoder	CDS	151775	152120	.	-	2	ID=cds.Gene.152834;Parent=Gene.152834
scaffold953	transdecoder	exon	151405	151555	.	-	.	ID=Gene.152834:exon3;Parent=Gene.152834
scaffold953	transdecoder	CDS	151405	151555	.	-	1	ID=cds.Gene.152834;Parent=Gene.152834
scaffold280	transdecoder	gene	5191	35374	.	-	.	ID=42541;Name=ORF
scaffold280	transdecoder	mRNA	5191	35374	.	-	.	ID=Gene.66485;Parent=42541;Name=ORF
scaffold280	transdecoder	exon	35161	35374	.	-	.	ID=Gene.66485:exon1;Parent=Gene.66485
scaffold280	transdecoder	CDS	35161	35374	.	-	0	ID=cds.Gene.66485;Parent=Gene.66485
scaffold280	transdecoder	exon	11482	11750	.	-	.	ID=Gene.66485:exon2;Parent=Gene.66485
scaffold280	transdecoder	CDS	11482	11750	.	-	2	ID=cds.Gene.66485;Parent=Gene.66485
scaffold280	transdecoder	exon	5191	6654	.	-	.	ID=Gene.66485:exon3;Parent=Gene.66485
scaffold280	transdecoder	CDS	5191	6654	.	-	0	ID=cds.Gene.66485;Parent=Gene.66485
scaffold280	transdecoder	gene	79541	79988	.	-	.	ID=42545;Name=ORF
scaffold280	transdecoder	mRNA	79541	79988	.	-	.	ID=Gene.66457;Parent=42545;Name=ORF
scaffold280	transdecoder	exon	79937	79988	.	-	.	ID=Gene.66457:exon1;Parent=Gene.66457
scaffold280	transdecoder	CDS	79937	79988	.	-	0	ID=cds.Gene.66457;Parent=Gene.66457
scaffold280	transdecoder	exon	79541	79713	.	-	.	ID=Gene.66457:exon2;Parent=Gene.66457
scaffold280	transdecoder	CDS	79541	79713	.	-	2	ID=cds.Gene.66457;Parent=Gene.66457
scaffold280	transdecoder	gene	190897	203842	.	-	.	ID=42551;Name=ORF
scaffold280	transdecoder	mRNA	190897	203842	.	-	.	ID=Gene.66474;Parent=42551;Name=ORF
scaffold280	transdecoder	exon	203191	203842	.	-	.	ID=Gene.66474:exon1;Parent=Gene.66474
scaffold280	transdecoder	CDS	203191	203842	.	-	0	ID=cds.Gene.66474;Parent=Gene.66474
scaffold280	transdecoder	exon	192744	192840	.	-	.	ID=Gene.66474:exon2;Parent=Gene.66474
scaffold280	transdecoder	CDS	192744	192840	.	-	2	ID=cds.Gene.66474;Parent=Gene.66474
scaffold280	transdecoder	exon	190897	191134	.	-	.	ID=Gene.66474:exon3;Parent=Gene.66474
scaffold280	transdecoder	CDS	190897	191134	.	-	1	ID=cds.Gene.66474;Parent=Gene.66474
scaffold280	transdecoder	gene	287594	309691	.	-	.	ID=42554;Name=ORF
scaffold280	transdecoder	mRNA	287594	309691	.	-	.	ID=Gene.66463;Parent=42554;Name=ORF
scaffold280	transdecoder	exon	309118	309691	.	-	.	ID=Gene.66463:exon1;Parent=Gene.66463
scaffold280	transdecoder	CDS	309118	309691	.	-	0	ID=cds.Gene.66463;Parent=Gene.66463

scaffold280	transdecoder	exon	294116	294163	.	-	.	ID=Gene.66463:exon2;Parent=Gene.66463
scaffold280	transdecoder	CDS	294116	294163	.	-	2	ID=cds.Gene.66463;Parent=Gene.66463
scaffold280	transdecoder	exon	287594	287910	.	-	.	ID=Gene.66463:exon3;Parent=Gene.66463
scaffold280	transdecoder	CDS	287594	287910	.	-	2	ID=cds.Gene.66463;Parent=Gene.66463
scaffold280	transdecoder	gene	356186	358265	.	+	.	ID=42562;Name=ORF
scaffold280	transdecoder	mRNA	356186	358265	.	+	.	ID=Gene.66489;Parent=42562;Name=ORF
scaffold280	transdecoder	five_prime_UTR	356186	356187	.	+	.	ID=Gene.66489:utr5p1;Parent=Gene.66489
scaffold280	transdecoder	exon	356186	356395	.	+	.	ID=Gene.66489:exon1;Parent=Gene.66489
scaffold280	transdecoder	CDS	356188	356395	.	+	0	ID=cds.Gene.66489;Parent=Gene.66489
scaffold280	transdecoder	exon	357770	357877	.	+	.	ID=Gene.66489:exon2;Parent=Gene.66489
scaffold280	transdecoder	CDS	357770	357828	.	+	2	ID=cds.Gene.66489;Parent=Gene.66489
scaffold280	transdecoder	exon	358011	358265	.	+	.	ID=Gene.66489:exon3;Parent=Gene.66489
scaffold280	transdecoder	three_prime_UTR	357829	357877	.	+	.	ID=Gene.66489:utr3p1;Parent=Gene.66489
scaffold280	transdecoder	three_prime_UTR	358011	358265	.	+	.	ID=Gene.66489:utr3p2;Parent=Gene.66489
scaffold826	transdecoder	gene	35588	51914	.	+	.	ID=90472;Name=ORF
scaffold826	transdecoder	mRNA	35588	51914	.	+	.	ID=Gene.142102;Parent=90472;Name=ORF
scaffold826	transdecoder	exon	35588	36113	.	+	.	ID=Gene.142102:exon1;Parent=Gene.142102
scaffold826	transdecoder	CDS	35588	36113	.	+	0	ID=cds.Gene.142102;Parent=Gene.142102
scaffold826	transdecoder	exon	51598	51914	.	+	.	ID=Gene.142102:exon2;Parent=Gene.142102
scaffold826	transdecoder	CDS	51598	51914	.	+	2	ID=cds.Gene.142102;Parent=Gene.142102
scaffold826	transdecoder	gene	152228	164599	.	+	.	ID=90477;Name=ORF
scaffold826	transdecoder	mRNA	152228	164599	.	+	.	ID=Gene.142103;Parent=90477;Name=ORF
scaffold826	transdecoder	exon	152228	152876	.	+	.	ID=Gene.142103:exon1;Parent=Gene.142103
scaffold826	transdecoder	CDS	152228	152876	.	+	0	ID=cds.Gene.142103;Parent=Gene.142103
scaffold826	transdecoder	exon	164286	164599	.	+	.	ID=Gene.142103:exon2;Parent=Gene.142103
scaffold826	transdecoder	CDS	164286	164599	.	+	2	ID=cds.Gene.142103;Parent=Gene.142103
scaffold826	transdecoder	gene	177257	177544	.	+	.	ID=90478;Name=ORF
scaffold826	transdecoder	mRNA	177257	177544	.	+	.	ID=Gene.142092;Parent=90478;Name=ORF
scaffold826	transdecoder	exon	177257	177544	.	+	.	ID=Gene.142092:exon1;Parent=Gene.142092
scaffold826	transdecoder	CDS	177257	177544	.	+	0	ID=cds.Gene.142092;Parent=Gene.142092
scaffold180	transdecoder	gene	331663	338240	.	-	.	ID=27360;Name=ORF
scaffold180	transdecoder	mRNA	331663	338240	.	-	.	ID=Gene.42259;Parent=27360;Name=ORF
scaffold180	transdecoder	exon	338143	338240	.	-	.	ID=Gene.42259:exon1;Parent=Gene.42259
scaffold180	transdecoder	CDS	338143	338240	.	-	0	ID=cds.Gene.42259;Parent=Gene.42259
scaffold180	transdecoder	exon	331663	331885	.	-	.	ID=Gene.42259:exon2;Parent=Gene.42259
scaffold180	transdecoder	CDS	331663	331885	.	-	1	ID=cds.Gene.42259;Parent=Gene.42259
scaffold180	transdecoder	gene	385874	388440	.	+	.	ID=27363;Name=ORF
scaffold180	transdecoder	mRNA	385874	388440	.	+	.	ID=Gene.42236;Parent=27363;Name=ORF
scaffold180	transdecoder	exon	385874	385889	.	+	.	ID=Gene.42236:exon1;Parent=Gene.42236
scaffold180	transdecoder	CDS	385874	385889	.	+	0	ID=cds.Gene.42236;Parent=Gene.42236
scaffold180	transdecoder	exon	388124	388440	.	+	.	ID=Gene.42236:exon2;Parent=Gene.42236
scaffold180	transdecoder	CDS	388124	388440	.	+	2	ID=cds.Gene.42236;Parent=Gene.42236
scaffold446	transdecoder	gene	1447	36506	.	+	.	ID=61595;Name=ORF
scaffold446	transdecoder	mRNA	1447	36506	.	+	.	ID=Gene.96883;Parent=61595;Name=ORF
scaffold446	transdecoder	five_prime_UTR	1447	1448	.	+	.	ID=Gene.96883:utr5p1;Parent=Gene.96883

scaffold446	transdecoder	exon	1447	1575	.	+	.	ID=Gene.96883:exon1;Parent=Gene.96883
scaffold446	transdecoder	CDS	1449	1575	.	+	0	ID=cds.Gene.96883;Parent=Gene.96883
scaffold446	transdecoder	exon	2062	2180	.	+	.	ID=Gene.96883:exon2;Parent=Gene.96883
scaffold446	transdecoder	CDS	2062	2180	.	+	2	ID=cds.Gene.96883;Parent=Gene.96883
scaffold446	transdecoder	exon	3375	3492	.	+	.	ID=Gene.96883:exon3;Parent=Gene.96883
scaffold446	transdecoder	CDS	3375	3492	.	+	0	ID=cds.Gene.96883;Parent=Gene.96883
scaffold446	transdecoder	exon	4683	4770	.	+	.	ID=Gene.96883:exon4;Parent=Gene.96883
scaffold446	transdecoder	CDS	4683	4770	.	+	2	ID=cds.Gene.96883;Parent=Gene.96883
scaffold446	transdecoder	exon	5330	5384	.	+	.	ID=Gene.96883:exon5;Parent=Gene.96883
scaffold446	transdecoder	CDS	5330	5384	.	+	1	ID=cds.Gene.96883;Parent=Gene.96883
scaffold446	transdecoder	exon	6542	6711	.	+	.	ID=Gene.96883:exon6;Parent=Gene.96883
scaffold446	transdecoder	CDS	6542	6711	.	+	0	ID=cds.Gene.96883;Parent=Gene.96883
scaffold446	transdecoder	exon	7955	8077	.	+	.	ID=Gene.96883:exon7;Parent=Gene.96883
scaffold446	transdecoder	CDS	7955	8077	.	+	1	ID=cds.Gene.96883;Parent=Gene.96883
scaffold446	transdecoder	exon	10580	10682	.	+	.	ID=Gene.96883:exon8;Parent=Gene.96883
scaffold446	transdecoder	CDS	10580	10682	.	+	1	ID=cds.Gene.96883;Parent=Gene.96883
scaffold446	transdecoder	exon	12203	12325	.	+	.	ID=Gene.96883:exon9;Parent=Gene.96883
scaffold446	transdecoder	CDS	12203	12325	.	+	0	ID=cds.Gene.96883;Parent=Gene.96883
scaffold446	transdecoder	exon	16469	16704	.	+	.	ID=Gene.96883:exon10;Parent=Gene.96883
scaffold446	transdecoder	CDS	16469	16704	.	+	0	ID=cds.Gene.96883;Parent=Gene.96883
scaffold446	transdecoder	exon	36407	36506	.	+	.	ID=Gene.96883:exon11;Parent=Gene.96883
scaffold446	transdecoder	CDS	36407	36506	.	+	1	ID=cds.Gene.96883;Parent=Gene.96883
scaffold446	transdecoder	gene	93457	93966	.	-	.	ID=61599;Name=ORF
scaffold446	transdecoder	mRNA	93457	93966	.	-	.	ID=Gene.96876;Parent=61599;Name=ORF
scaffold446	transdecoder	exon	93457	93966	.	-	.	ID=Gene.96876:exon1;Parent=Gene.96876
scaffold446	transdecoder	CDS	93457	93966	.	-	0	ID=cds.Gene.96876;Parent=Gene.96876
scaffold446	transdecoder	gene	253892	265153	.	+	.	ID=61603;Name=ORF
scaffold446	transdecoder	mRNA	253892	265153	.	+	.	ID=Gene.96879;Parent=61603;Name=ORF
scaffold446	transdecoder	exon	253892	254381	.	+	.	ID=Gene.96879:exon1;Parent=Gene.96879
scaffold446	transdecoder	CDS	253892	254381	.	+	0	ID=cds.Gene.96879;Parent=Gene.96879
scaffold446	transdecoder	exon	259760	259763	.	+	.	ID=Gene.96879:exon2;Parent=Gene.96879
scaffold446	transdecoder	CDS	259760	259763	.	+	2	ID=cds.Gene.96879;Parent=Gene.96879
scaffold446	transdecoder	exon	265051	265153	.	+	.	ID=Gene.96879:exon3;Parent=Gene.96879
scaffold446	transdecoder	CDS	265051	265153	.	+	1	ID=cds.Gene.96879;Parent=Gene.96879
scaffold446	transdecoder	gene	287411	312650	.	-	.	ID=61604;Name=ORF
scaffold446	transdecoder	mRNA	287411	312650	.	-	.	ID=Gene.96875;Parent=61604;Name=ORF
scaffold446	transdecoder	exon	312529	312650	.	-	.	ID=Gene.96875:exon1;Parent=Gene.96875
scaffold446	transdecoder	CDS	312529	312650	.	-	0	ID=cds.Gene.96875;Parent=Gene.96875
scaffold446	transdecoder	exon	309487	309568	.	-	.	ID=Gene.96875:exon2;Parent=Gene.96875
scaffold446	transdecoder	CDS	309487	309568	.	-	1	ID=cds.Gene.96875;Parent=Gene.96875
scaffold446	transdecoder	exon	307970	308181	.	-	.	ID=Gene.96875:exon3;Parent=Gene.96875
scaffold446	transdecoder	CDS	307970	308181	.	-	0	ID=cds.Gene.96875;Parent=Gene.96875
scaffold446	transdecoder	exon	305008	305144	.	-	.	ID=Gene.96875:exon4;Parent=Gene.96875
scaffold446	transdecoder	CDS	305008	305144	.	-	1	ID=cds.Gene.96875;Parent=Gene.96875
scaffold446	transdecoder	exon	304331	304484	.	-	.	ID=Gene.96875:exon5;Parent=Gene.96875

scaffold446	transdecoder	CDS	304331	304484	.	-	2	ID=cds.Gene.96875;Parent=Gene.96875
scaffold446	transdecoder	exon	300493	300748	.	-	.	ID=Gene.96875:exon6;Parent=Gene.96875
scaffold446	transdecoder	CDS	300493	300748	.	-	1	ID=cds.Gene.96875;Parent=Gene.96875
scaffold446	transdecoder	exon	299911	299996	.	-	.	ID=Gene.96875:exon7;Parent=Gene.96875
scaffold446	transdecoder	CDS	299911	299996	.	-	0	ID=cds.Gene.96875;Parent=Gene.96875
scaffold446	transdecoder	exon	298230	298348	.	-	.	ID=Gene.96875:exon8;Parent=Gene.96875
scaffold446	transdecoder	CDS	298230	298348	.	-	1	ID=cds.Gene.96875;Parent=Gene.96875
scaffold446	transdecoder	exon	294933	295111	.	-	.	ID=Gene.96875:exon9;Parent=Gene.96875
scaffold446	transdecoder	CDS	294933	295111	.	-	2	ID=cds.Gene.96875;Parent=Gene.96875
scaffold446	transdecoder	exon	294231	294350	.	-	.	ID=Gene.96875:exon10;Parent=Gene.96875
scaffold446	transdecoder	CDS	294231	294350	.	-	0	ID=cds.Gene.96875;Parent=Gene.96875
scaffold446	transdecoder	exon	289799	290668	.	-	.	ID=Gene.96875:exon11;Parent=Gene.96875
scaffold446	transdecoder	CDS	289799	290668	.	-	0	ID=cds.Gene.96875;Parent=Gene.96875
scaffold446	transdecoder	exon	289484	289676	.	-	.	ID=Gene.96875:exon12;Parent=Gene.96875
scaffold446	transdecoder	CDS	289484	289676	.	-	0	ID=cds.Gene.96875;Parent=Gene.96875
scaffold446	transdecoder	exon	288577	288670	.	-	.	ID=Gene.96875:exon13;Parent=Gene.96875
scaffold446	transdecoder	CDS	288577	288670	.	-	2	ID=cds.Gene.96875;Parent=Gene.96875
scaffold446	transdecoder	exon	287411	287447	.	-	.	ID=Gene.96875:exon14;Parent=Gene.96875
scaffold446	transdecoder	CDS	287411	287447	.	-	1	ID=cds.Gene.96875;Parent=Gene.96875
scaffold1633	transdecoder	gene	67788	97155	.	+	.	ID=22876;Name=ORF
scaffold1633	transdecoder	mRNA	67788	97155	.	+	.	ID=Gene.35389;Parent=22876;Name=ORF
scaffold1633	transdecoder	exon	67788	67877	.	+	.	ID=Gene.35389:exon1;Parent=Gene.35389
scaffold1633	transdecoder	CDS	67788	67877	.	+	0	ID=cds.Gene.35389;Parent=Gene.35389
scaffold1633	transdecoder	exon	69478	69541	.	+	.	ID=Gene.35389:exon2;Parent=Gene.35389
scaffold1633	transdecoder	CDS	69478	69541	.	+	0	ID=cds.Gene.35389;Parent=Gene.35389
scaffold1633	transdecoder	exon	70167	70219	.	+	.	ID=Gene.35389:exon3;Parent=Gene.35389
scaffold1633	transdecoder	CDS	70167	70219	.	+	2	ID=cds.Gene.35389;Parent=Gene.35389
scaffold1633	transdecoder	exon	71195	71287	.	+	.	ID=Gene.35389:exon4;Parent=Gene.35389
scaffold1633	transdecoder	CDS	71195	71287	.	+	0	ID=cds.Gene.35389;Parent=Gene.35389
scaffold1633	transdecoder	exon	73198	73290	.	+	.	ID=Gene.35389:exon5;Parent=Gene.35389
scaffold1633	transdecoder	CDS	73198	73290	.	+	0	ID=cds.Gene.35389;Parent=Gene.35389
scaffold1633	transdecoder	exon	73886	74053	.	+	.	ID=Gene.35389:exon6;Parent=Gene.35389
scaffold1633	transdecoder	CDS	73886	74053	.	+	0	ID=cds.Gene.35389;Parent=Gene.35389
scaffold1633	transdecoder	exon	75018	75081	.	+	.	ID=Gene.35389:exon7;Parent=Gene.35389
scaffold1633	transdecoder	CDS	75018	75081	.	+	0	ID=cds.Gene.35389;Parent=Gene.35389
scaffold1633	transdecoder	exon	75554	75601	.	+	.	ID=Gene.35389:exon8;Parent=Gene.35389
scaffold1633	transdecoder	CDS	75554	75601	.	+	2	ID=cds.Gene.35389;Parent=Gene.35389
scaffold1633	transdecoder	exon	75911	76006	.	+	.	ID=Gene.35389:exon9;Parent=Gene.35389
scaffold1633	transdecoder	CDS	75911	76006	.	+	2	ID=cds.Gene.35389;Parent=Gene.35389
scaffold1633	transdecoder	exon	78550	78636	.	+	.	ID=Gene.35389:exon10;Parent=Gene.35389
scaffold1633	transdecoder	CDS	78550	78636	.	+	2	ID=cds.Gene.35389;Parent=Gene.35389
scaffold1633	transdecoder	exon	89972	90024	.	+	.	ID=Gene.35389:exon11;Parent=Gene.35389
scaffold1633	transdecoder	CDS	89972	90024	.	+	2	ID=cds.Gene.35389;Parent=Gene.35389
scaffold1633	transdecoder	exon	90545	90670	.	+	.	ID=Gene.35389:exon12;Parent=Gene.35389
scaffold1633	transdecoder	CDS	90545	90670	.	+	0	ID=cds.Gene.35389;Parent=Gene.35389

scaffold1633	transdecoder	exon	91028	91091	.	+	.	ID=Gene.35389:exon13;Parent=Gene.35389
scaffold1633	transdecoder	CDS	91028	91091	.	+	0	ID=cds.Gene.35389;Parent=Gene.35389
scaffold1633	transdecoder	exon	92004	92424	.	+	.	ID=Gene.35389:exon14;Parent=Gene.35389
scaffold1633	transdecoder	CDS	92004	92424	.	+	2	ID=cds.Gene.35389;Parent=Gene.35389
scaffold1633	transdecoder	exon	92469	92806	.	+	.	ID=Gene.35389:exon15;Parent=Gene.35389
scaffold1633	transdecoder	CDS	92469	92806	.	+	1	ID=cds.Gene.35389;Parent=Gene.35389
scaffold1633	transdecoder	exon	92945	93058	.	+	.	ID=Gene.35389:exon16;Parent=Gene.35389
scaffold1633	transdecoder	CDS	92945	93058	.	+	2	ID=cds.Gene.35389;Parent=Gene.35389
scaffold1633	transdecoder	exon	93331	93587	.	+	.	ID=Gene.35389:exon17;Parent=Gene.35389
scaffold1633	transdecoder	CDS	93331	93587	.	+	2	ID=cds.Gene.35389;Parent=Gene.35389
scaffold1633	transdecoder	exon	96796	97155	.	+	.	ID=Gene.35389:exon18;Parent=Gene.35389
scaffold1633	transdecoder	CDS	96796	97155	.	+	0	ID=cds.Gene.35389;Parent=Gene.35389
scaffold1633	transdecoder	gene	119547	127110	.	-	.	ID=22880;Name=ORF
scaffold1633	transdecoder	mRNA	119547	127110	.	-	.	ID=Gene.35372;Parent=22880;Name=ORF
scaffold1633	transdecoder	exon	127014	127110	.	-	.	ID=Gene.35372:exon1;Parent=Gene.35372
scaffold1633	transdecoder	CDS	127014	127110	.	-	0	ID=cds.Gene.35372;Parent=Gene.35372
scaffold1633	transdecoder	exon	125833	126138	.	-	.	ID=Gene.35372:exon2;Parent=Gene.35372
scaffold1633	transdecoder	CDS	125833	126138	.	-	2	ID=cds.Gene.35372;Parent=Gene.35372
scaffold1633	transdecoder	exon	124913	125042	.	-	.	ID=Gene.35372:exon3;Parent=Gene.35372
scaffold1633	transdecoder	CDS	124913	125042	.	-	2	ID=cds.Gene.35372;Parent=Gene.35372
scaffold1633	transdecoder	exon	124151	124620	.	-	.	ID=Gene.35372:exon4;Parent=Gene.35372
scaffold1633	transdecoder	CDS	124151	124620	.	-	1	ID=cds.Gene.35372;Parent=Gene.35372
scaffold1633	transdecoder	exon	120534	120679	.	-	.	ID=Gene.35372:exon5;Parent=Gene.35372
scaffold1633	transdecoder	CDS	120534	120679	.	-	2	ID=cds.Gene.35372;Parent=Gene.35372
scaffold1633	transdecoder	exon	119547	119978	.	-	.	ID=Gene.35372:exon6;Parent=Gene.35372
scaffold1633	transdecoder	CDS	119547	119978	.	-	0	ID=cds.Gene.35372;Parent=Gene.35372
scaffold1633	transdecoder	gene	136037	153238	.	-	.	ID=22882;Name=ORF
scaffold1633	transdecoder	mRNA	136037	153238	.	-	.	ID=Gene.35387;Parent=22882;Name=ORF
scaffold1633	transdecoder	exon	153080	153238	.	-	.	ID=Gene.35387:exon1;Parent=Gene.35387
scaffold1633	transdecoder	CDS	153080	153238	.	-	0	ID=cds.Gene.35387;Parent=Gene.35387
scaffold1633	transdecoder	exon	151166	151244	.	-	.	ID=Gene.35387:exon2;Parent=Gene.35387
scaffold1633	transdecoder	CDS	151166	151244	.	-	0	ID=cds.Gene.35387;Parent=Gene.35387
scaffold1633	transdecoder	exon	149540	149680	.	-	.	ID=Gene.35387:exon3;Parent=Gene.35387
scaffold1633	transdecoder	CDS	149540	149680	.	-	2	ID=cds.Gene.35387;Parent=Gene.35387
scaffold1633	transdecoder	exon	149122	149206	.	-	.	ID=Gene.35387:exon4;Parent=Gene.35387
scaffold1633	transdecoder	CDS	149122	149206	.	-	2	ID=cds.Gene.35387;Parent=Gene.35387
scaffold1633	transdecoder	exon	147966	148076	.	-	.	ID=Gene.35387:exon5;Parent=Gene.35387
scaffold1633	transdecoder	CDS	147966	148076	.	-	1	ID=cds.Gene.35387;Parent=Gene.35387
scaffold1633	transdecoder	exon	143898	144000	.	-	.	ID=Gene.35387:exon6;Parent=Gene.35387
scaffold1633	transdecoder	CDS	143898	144000	.	-	1	ID=cds.Gene.35387;Parent=Gene.35387
scaffold1633	transdecoder	exon	143265	143381	.	-	.	ID=Gene.35387:exon7;Parent=Gene.35387
scaffold1633	transdecoder	CDS	143265	143381	.	-	0	ID=cds.Gene.35387;Parent=Gene.35387
scaffold1633	transdecoder	exon	140093	140174	.	-	.	ID=Gene.35387:exon8;Parent=Gene.35387
scaffold1633	transdecoder	CDS	140093	140174	.	-	0	ID=cds.Gene.35387;Parent=Gene.35387
scaffold1633	transdecoder	exon	139031	139110	.	-	.	ID=Gene.35387:exon9;Parent=Gene.35387

scaffold1633	transdecoder	CDS	139031	139110	.	-	2	ID=cds.Gene.35387;Parent=Gene.35387
scaffold1633	transdecoder	exon	136037	136228	.	-	.	ID=Gene.35387:exon10;Parent=Gene.35387
scaffold1633	transdecoder	CDS	136037	136228	.	-	0	ID=cds.Gene.35387;Parent=Gene.35387
scaffold1276	transdecoder	gene	47193	50809	.	+	.	ID=11425;Name=ORF
scaffold1276	transdecoder	mRNA	47193	50809	.	+	.	ID=Gene.17656;Parent=11425;Name=ORF
scaffold1276	transdecoder	exon	47193	47712	.	+	.	ID=Gene.17656:exon1;Parent=Gene.17656
scaffold1276	transdecoder	CDS	47193	47712	.	+	0	ID=cds.Gene.17656;Parent=Gene.17656
scaffold1276	transdecoder	exon	49403	49560	.	+	.	ID=Gene.17656:exon2;Parent=Gene.17656
scaffold1276	transdecoder	CDS	49403	49560	.	+	2	ID=cds.Gene.17656;Parent=Gene.17656
scaffold1276	transdecoder	exon	50486	50809	.	+	.	ID=Gene.17656:exon3;Parent=Gene.17656
scaffold1276	transdecoder	CDS	50486	50809	.	+	0	ID=cds.Gene.17656;Parent=Gene.17656
scaffold1276	transdecoder	gene	85669	103137	.	+	.	ID=11428;Name=ORF
scaffold1276	transdecoder	mRNA	85669	103137	.	+	.	ID=Gene.17658;Parent=11428;Name=ORF
scaffold1276	transdecoder	exon	85669	86161	.	+	.	ID=Gene.17658:exon1;Parent=Gene.17658
scaffold1276	transdecoder	CDS	85669	86161	.	+	0	ID=cds.Gene.17658;Parent=Gene.17658
scaffold1276	transdecoder	exon	102683	103137	.	+	.	ID=Gene.17658:exon2;Parent=Gene.17658
scaffold1276	transdecoder	CDS	102683	103137	.	+	2	ID=cds.Gene.17658;Parent=Gene.17658
scaffold1276	transdecoder	gene	108163	111679	.	+	.	ID=11429;Name=ORF
scaffold1276	transdecoder	mRNA	108163	111679	.	+	.	ID=Gene.17661;Parent=11429;Name=ORF
scaffold1276	transdecoder	exon	108163	108300	.	+	.	ID=Gene.17661:exon1;Parent=Gene.17661
scaffold1276	transdecoder	CDS	108163	108300	.	+	0	ID=cds.Gene.17661;Parent=Gene.17661
scaffold1276	transdecoder	exon	108515	108615	.	+	.	ID=Gene.17661:exon2;Parent=Gene.17661
scaffold1276	transdecoder	CDS	108515	108615	.	+	0	ID=cds.Gene.17661;Parent=Gene.17661
scaffold1276	transdecoder	exon	109100	109217	.	+	.	ID=Gene.17661:exon3;Parent=Gene.17661
scaffold1276	transdecoder	CDS	109100	109217	.	+	1	ID=cds.Gene.17661;Parent=Gene.17661
scaffold1276	transdecoder	exon	109449	109498	.	+	.	ID=Gene.17661:exon4;Parent=Gene.17661
scaffold1276	transdecoder	CDS	109449	109498	.	+	0	ID=cds.Gene.17661;Parent=Gene.17661
scaffold1276	transdecoder	exon	110250	110439	.	+	.	ID=Gene.17661:exon5;Parent=Gene.17661
scaffold1276	transdecoder	CDS	110250	110439	.	+	1	ID=cds.Gene.17661;Parent=Gene.17661
scaffold1276	transdecoder	exon	110741	110797	.	+	.	ID=Gene.17661:exon6;Parent=Gene.17661
scaffold1276	transdecoder	CDS	110741	110797	.	+	0	ID=cds.Gene.17661;Parent=Gene.17661
scaffold1276	transdecoder	exon	111052	111132	.	+	.	ID=Gene.17661:exon7;Parent=Gene.17661
scaffold1276	transdecoder	CDS	111052	111132	.	+	0	ID=cds.Gene.17661;Parent=Gene.17661
scaffold1276	transdecoder	exon	111371	111679	.	+	.	ID=Gene.17661:exon8;Parent=Gene.17661
scaffold1276	transdecoder	CDS	111371	111679	.	+	0	ID=cds.Gene.17661;Parent=Gene.17661

Supplementary Table 1

Gene Name	2hpf	4hpf	6hpf	8hpf	13hpf	18hpf	23hpf	26hpf	27hpf	30hpf	36hpf	48hpf	54hpf	60hpf	72hpf	84hpf
Hox1	0.171201	0.511965	1.51738	0	28.6844	30.8001	7.76521	14.8163	6.5041	6.08023	12.3281	7.00236	7.16333	13.1674	13.6035	14.6324
Hox2	0.761162	0.830919	0.153618	0.287199	0.172314	9.79244	19.8087	26.316	6.26163	2.98155	5.72044	2.50905	1.70803	4.89708	5.09231	5.46748
Hox3	0.871596	1.69144	0	0	0	0	0	0.664438	0.432237	0	3.93071	0.576672	2.24315	0.49431	2.0662	0
Hox4	0	0	0.473597	0.196339	65.6164	35.3547	9.41803	18.9779	9.67464	5.69592	48.2808	9.78839	9.08917	34.3133	31.1308	25.4243
Hox5	0	0	0	0.414374	0	0	0.148363	0.230293	1.14313	0.111336	1.26658	0	0.59958	0.364755	0.99578	0.42368
Lox5	0	0	0.486358	0	14.8949	46.3018	15.0134	10.1943	5.70952	3.03321	9.8611	1.62441	3.79211	6.47783	4.5803	3.43496
Lox4	0	0	0	0	1.04993	4.40817	6.84024	10.6252	4.79951	2.00322	12.9751	3.63628	2.24748	10.2473	15.8881	8.42197
Lox2	0	0	0	0	0	0	0	0	0	0	0.464894	1.37844	2.32229	3.57047	3.62816	2.8405
Post2	0	0.24766	0.275594	1.02603	198.836	119.598	77.6776	158.209	130.492	63.2854	139.221	43.7139	33.6725	113.673	80.3193	57.0943
Post1	0	0	0	0	1.11113	1.85571	3.52344	10.7707	15.5632	11.4728	55.8559	14.6378	20.1796	30.1239	37.3983	27.7983
Gsx	0	0	0	0.386372	0	0.347798	0	0	0	0	0	0	0	0	0	0
Xlox	0	0	0	0	0	0.184675	0.881016	1.13956	3.16778	2.75472	9.47121	5.57624	5.43959	7.58132	8.95903	6.00995
Cdx	0.689311	1.12868	0.387011	18.2354	97.5659	48.9117	31.5202	38.8104	30.7053	15.2073	32.7694	16.754	13.4254	33.7677	29.9453	25.0949

Supplementary Table 2

	Primers	
Genes	Forward	Reverse
Dro-Hox1	TATCAGCGATCACCTGTGACAG	CACAACGCCATGGAAGTAACAC
Dro-Hox2	CGGTACCGGATATGACTGGATG	GAATCGTCCGAAGCATTGTCC
Dro-Hox3	TGAGTCCGAACCATTACGATGA	GGACATGTTACCGTCCAGCTT
Dro-Hox4	AATGGATTCACTTCGCCGACG	TGCTCATATGGTGTCCGATCTG
Dro-Lox5	TGTGTTACGACCATGAAGCAATG	ATGTCATCTCACGTGGTCTATCG
Dro-Lox2	GGACTACCGGTCATCGAGGTATT	TTCCTTAATCGCCTGCAGTTCTT
Dro-Lox4	TCTAGTGAGAATCCGCATGTTGG	AGTGTGCCACTTCTATTCTTCGT
Dro-Post2	ACATGTCGCCGCTTAATCCTAT	AAGTGTTGTTGTGACAGTGAGTG
Dro-Xlox	GATCTTGCGCCTATTACAACCAG	CAAGGCAGCATCAATGTACACAA

Supplementary Table 3

Phylum	Class	Species	Code name	GeneBank
Mollusca	Bivalvia	*Nucula tumidula*	Nucula_tumidula_Hox1	APD15698
			Nucula_tumidula_Hox2	APD15699
			Nucula_tumidula_Hox3	APD15700
			Nucula_tumidula_Hox4	APD15701
			Nucula_tumidula_Lox5	APD15702
			Nucula_tumidula_Lox4	APD15703
			Nucula_tumidula_Post1	APD15704
			Nucula_tumidula_Post2	APD15705
			Nucula_tumidula_Cdx	APD15706
			Nucula_tumidula_Xlox	APD15707
Mollusca	Gastropoda	*Gibbula varia*	Gibbula_varia_Hox1	ACX84671
			Gibbula_varia_Hox2	ADJ18233
			Gibbula_varia_Hox3	ADJ18232
			Gibbula_varia_Hox4	ACX84672
			Gibbula_varia_Hox5	ADJ18234
			Gibbula_varia_Lox5	ADJ18235
			Gibbula_varia_Lox4	ADJ18237
			Gibbula_varia_Lox2	ADJ18238
			Gibbula_varia_Post1	ACX84673
			Gibbula_varia_Post2	ACX84674
			Gibbula_varia_Gsx	ALM30866
			Gibbula_varia_Xlox	ADJ18240
Mollusca	Polyplacophora	*Acanthochitona crinita*	Acanthochitona_crinita_Hox1	APD15641
			Acanthochitona_crinita_Hox2	APD15642
			Acanthochitona_crinita_Hox3	APD15643
			Acanthochitona_crinita_Hox4	APD15644
			Acanthochitona_crinita_Hox5	APD15645
			Acanthochitona_crinita_Lox5	APD15646

			Acanthochitona_crinita_Lox4	APD15647
			Acanthochitona_crinita_Lox2	APD15648
			Acanthochitona_crinita_Post2	APD15649
			Acanthochitona_crinita_Cdx	APD15650
Annelida	Polychaeta	*Alitta virens*	Alitta_virens_Hox1	AAD46166
			Alitta_virens_Hox2	AAD46167
			Alitta_virens_Hox3	AAD46168
			Alitta_virens_Hox4	AAD46169
			Alitta_virens_Hox5	AAD46170
			Alitta_virens_Lox5	AAD46174
			Alitta_virens_Lox2	AAD46171
			Alitta_virens_Post1	AAD46175
			Alitta_virens_Post2	AAD46176
			Alitta_virens_Gsx	ABB59695
Chordata	Actinopterygii	*Lepisosteus oculatus*	Lepisosteus_oculatus_Gsx	XP_006627824
Echinodermata	Asteroidea	*Patiria miniata*	Patiria_miniata_Gsx	AGK89736
Priapulida	Priapulimorpha	*Priapulus caudatus*	Priapulus_caudatus_Gsx	XP_014665932
Annelida	Polychaeta	*Capitella teleta*	Capitella_telleta_Gsx	AAZ23124
Hemichordata	Enteropneusta	*Ptychodera flava*	Ptychodera_flava_Gsx	AAR07642
Chordata	Leptocardii	*Branchiostoma floridae*	Branchiostoma_floridae_Cdx	AAC39017
Echinodermata	Echinoidea	*Strongylocentrotus purpuratus*	Strongylocentrotus_purpuratus_Xlox	AAN17337
Annelida	Polychaeta	*Platynereis dumerilii*	Platynereis_dumerilii_Hox3	ABD04656
			Platynereis_dumerilii_Hox4	ABD04658
			Platynereis_dumerilii_Lox5	ABD04654
			Platynereis_dumerilii_Lox2	ABD04659
			Platynereis_dumerilii_Post1	ABD04653
			Platynereis_dumerilii_Post2	ABD04651
			Platynereis_dumerilii_Dls	CAJ38799
			Platynereis_dumerilii_Eng	CAE46753
			Platynereis_dumerilii_Xlox	ACH87551
			Platynereis_dumerilii_Cdx	ACH87546
Brachiopoda	Lingulata	*Lingula anatina*	Lingula_anatina_Hox1	AAD45587

			Lingula_anatina_Hox3	AAD45588
			Lingula_anatina_Hox5	AAD45589
			Lingula_anatina_Lox5	AAD45591
			Lingula_anatina_Lox4	AAD45593
			Lingula_anatina_Lox2	AAD45592
			Lingula_anatina_Post1	AAD45594
			Lingula_anatina_Post2	AAD45595
Mollusca	Neomeniomorpha	*Wirenia argentea*	Wirenia_argentea_Post1	APD15718
			Wirenia_argentea_Post2	APD15719
			Wirenia_argentea_Hox3	APD15711
			Wirenia_argentea_Hox4	APD15712
			Wirenia_argentea_Hox5	APD15713
			Wirenia_argentea_Lox5	APD15714
			Wirenia_argentea_Lox2	APD15716
Mollusca	Bivalvia	*Crassostrea gigas*	Crassostrea_gigas_Hox1	EKC32705
			Crassostrea_gigas_Hox2	EKC32708
			Crassostrea_gigas_Hox3	EKC32709
			Crassostrea_gigas_Hox4	EKC32713
			Crassostrea_gigas_Lox5	EKC41105
			Crassostrea_gigas_Lox4	EKC41102
			Crassostrea_gigas_Lox2	EKC39601
			Crassostrea_gigas_Post1	EKC29602
			Crassostrea_gigas_Post2	EKC29599
			Crassostrea_gigas_Xlox	XP_011426134
			Crassostrea_gigas_Cdx	XP_011420260
			Crassostrea_gigas_Gsx	XP_011426138
Chordata	Ascidiacea	*Ciona intestinalis*	Ciona_intestinalis_Hox1	NP_001122333
			Ciona_intestinalis_Hox2	CAD59668
			Ciona_intestinalis_Hox4	NP_001027781
			Ciona_intestinalis_Hox5	NP_001027665
			Ciona_intestinalis_Hox6	CAD59670
Arthropoda	Insecta	*Drosophila melanogaster*	Drosophila_melanogaster_Hox1	CAB57787

			Drosophila_melanogaster_Hox2	CAA45271
			Drosophila_melanogaster_Hox3	P09089
			Drosophila_melanogaster_Hox4	P07548
			Drosophila_melanogaster_Hox5	NP_524248
			Drosophila_melanogaster_Dls	NP_726486
			Drosophila_melanogaster_Eng	P02836
			Drosophila_melanogaster_Cdx	NP_599128
			Drosophila_melanogaster_Gsx	NP_996087
Hemichordata	Enteropneusta	*Saccoglossus kowalevskii*	Saccoglossus_kowalevskii_Hox1	AAP79296
			Saccoglossus_kowalevskii_Hox2	ABK00018
			Saccoglossus_kowalevskii_Hox3	AAP79286
			Saccoglossus_kowalevskii_Hox4	AAP79297
			Saccoglossus_kowalevskii_Hox5	ABK00019
			Saccoglossus_kowalevskii_Hox6	ABK00020
Mollusca	Cephalopoda	*Euprymna scolopes*	Euprymna_scolopes_Hox1	AAL25804
			Euprymna_scolopes_Hox3	AAR16188
			Euprymna_scolopes_Hox5	AAR16189
			Euprymna_scolopes_Lox4	AAL25810
			Euprymna_scolopes_Post1	AAL25811
			Euprymna_scolopes_Post2	AAL25812
			Euprymna_scolopes_Xlox	ABD16192
			Euprymna_scolopes_Gsx	AAV85466
Mollusca	Cephalopoda	*Idiosepius notoides*	Idiosepius_notoides_Hox3	APD15688
			Idiosepius_notoides_Hox5	APD15689
			Idiosepius_notoides_Lox5	APD15686
			Idiosepius_notoides_Lox4	APD15720
			Idiosepius_notoides_Post2	APD15690
Mollusca	Gastropoda	*Haliotis asinina*	Haliotis_asinina_Hox3	AAK17185
			Haliotis_asinina_Hox4	AAK11240
			Haliotis_asinina_Hox5	AAF78248
Mollusca	Scaphopoda	*Antalis entalis*	Antalis_entalis_Hox1	APD15651
			Antalis_entalis_Hox2	APD15652

			Antalis_entalis_Hox3	APD15653
			Antalis_entalis_Hox4	APD15654
			Antalis_entalis_Hox5	APD15655
			Antalis_entalis_Lox5	APD15656
			Antalis_entalis_Lox4	APD15658
			Antalis_entalis_Lox2	APD15657
			Antalis_entalis_Post1	APD15659
			Antalis_entalis_Post2	APD15660
			Antalis_entalis_Cdx	APD15661
			Antalis_entalis_Gsx	APD15662
Mollusca	Solenogastres	*Gymnomenia pellucida*	Gymnomenia_pellucida_Hox1	APD15663
			Gymnomenia_pellucida_Hox2	APD15664
			Gymnomenia_pellucida_Hox3	APD15665
			Gymnomenia_pellucida_Hox4	APD15666
			Gymnomenia_pellucida_Hox5	APD15667
			Gymnomenia_pellucida_Lox5	APD15668
			Gymnomenia_pellucida_Lox4	APD15670
			Gymnomenia_pellucida_Lox2	APD15671
			Gymnomenia_pellucida_Post1	APD15672
			Gymnomenia_pellucida_Post2	APD15673
			Gymnomenia_pellucida_Cdx	APD15674
			Gymnomenia_pellucida_Gsx	APD15675

Supplementary Table 4

Scaffolds	Genes	Size
953	*Dro-Hox1*	*232,15 Kb*
280	*Dro-Hox2*<br>*Dro-Hox3*<br>*Dro-Hox4*<br>*Dro-Hox5*	*369,15 Kb*
826	*Dro-Lox4*<br>*Dro-Lox5*	*246,17 Kb*
180	*Dro-Lox2*	*421,98 Kb*
446	*Dro-Post1*<br>*Dro-Post2*	*314,96 Kb*
1633	*Dro-Cdx*	*177,01 Kb*
1276	*Dro-Gsx*<br>*Dro-Xlox*	*199,78 Kb*
